# Fluorescent Probes *cis*- and *trans*-Parinaric Acids in Fluid and Gel Lipid Bilayers: A Molecular Dynamics Study

**DOI:** 10.3390/molecules28052241

**Published:** 2023-02-28

**Authors:** Alexandre C. Oliveira, Hugo A. L. Filipe, Luís M. S. Loura

**Affiliations:** 1 Coimbra Chemistry Center, Institute of Molecular Sciences (CQC-IMS), University of Coimbra, 3004-535 Coimbra, Portugal; 2 Department of Chemistry, Faculty of Sciences and Technology, University of Coimbra, 3004-535 Coimbra, Portugal; 3 Polytechnic of Guarda, CPIRN-IPG—Center of Potential and Innovation of Natural Resources, 6300-559 Guarda, Portugal; 4 Faculty of Pharmacy, University of Coimbra, 3000-548 Coimbra, Portugal; 5 CNC—Center for Neuroscience and Cell Biology, University of Coimbra, 3004-535 Coimbra, Portugal

**Keywords:** fluorescence spectroscopy, lipid membranes, membrane probe, molecular dynamics simulations

## Abstract

Fluorescence probes are indispensable tools in biochemical and biophysical membrane studies. Most of them possess extrinsic fluorophores, which often constitute a source of uncertainty and potential perturbation to the host system. In this regard, the few available intrinsically fluorescent membrane probes acquire increased importance. Among them, *cis*- and *trans*-parinaric acids (*c*-PnA and *t*-PnA, respectively) stand out as probes of membrane order and dynamics. These two compounds are long-chained fatty acids, differing solely in the configurations of two double bonds of their conjugated tetraene fluorophore. In this work, we employed all-atom and coarse-grained molecular dynamics simulations to study the behavior of *c*-PnA and *t*-PnA in lipid bilayers of 1-palmitoyl-2-oleoyl-*sn*-glycero-3-phosphocholine (POPC) and 1,2-dipalmitoyl-*sn*-glycero-3-phosphocholine (DPPC), representative of the liquid disordered and solid ordered lipid phases, respectively. All-atom simulations indicate that the two probes show similar location and orientation in the simulated systems, with the carboxylate facing the water/lipid interface and the tail spanning the membrane leaflet. The two probes establish interactions with the solvent and lipids to a similar degree in POPC. However, the almost linear *t*-PnA molecules have tighter lipid packing around them, especially in DPPC, where they also interact more with positively charged lipid choline groups. Probably for these reasons, while both probes show similar partition (assessed from computed free energy profiles across bilayers) to POPC, *t*-PnA clearly partitions more extensively than *c*-PnA to the gel phase. *t*-PnA also displays more hindered fluorophore rotation, especially in DPPC. Our results agree very well with experimental fluorescence data from the literature and allow deeper understanding of the behavior of these two reporters of membrane organization.

## 1. Introduction

Fluorescence-based spectroscopic and microscopic methods are tools of paramount importance in biophysical and biochemical research [1], and in membrane biophysical studies in particular [2,3,4]. Fluorescence possesses unrivalled sensitivity, often nanomolar or even below (such as in fluorescence correlation spectroscopy). It provides a number of different observables (excitation and emission spectra, steady-state and time-resolved intensity and anisotropy, quenching, Förster resonance energy transfer), which may be used for multiparametric studies, allowing insights on phenomena as diverse as partition to and location within the membrane, translocation and permeation, lateral and rotational diffusion, aggregation of identical or different membrane-associated species, lateral compartmentalization and phase separation, to name some of the most important ones.

However, fluorescence emission requires the existence of convenient fluorophores. While most proteins exhibit natural fluorescence due to the presence of aromatic residues, this is not typically the case with membranes, which are, for this reason, often labeled with extrinsic probes [1]. Many such fluorescent reporters have been synthesized and are commercially available, with a wide variety of fluorophores. While this allows for labeling membranes with probes of desirable photophysical properties (such as high quantum yield, photostability, microenvironmental sensitivity and visible/near-infrared emission), the use of such reporters always raises important concerns, namely: what region of the bilayer they are reporting on (i.e., where are they located within the membrane) and their orientation/configuration when inserted; and what is the extent and range of perturbation they cause to host membrane properties [5]. To gain insight on these issues, a methodology that allows independent and detailed monitoring of both probe and host lipid(s) is required. In the past two decades, molecular dynamics (MD) simulations have come to the fore as tools to unravel the peculiarities of behavior of fluorescent membrane probes, with unique detail [6,7,8,9]. The tremendous increase in computational power available for researchers, together with the continuous improvement in force field accuracy and MD algorithm efficiency, have made this technique an important ally of fluorescence-based membrane biophysicists.

**Figure 1:**
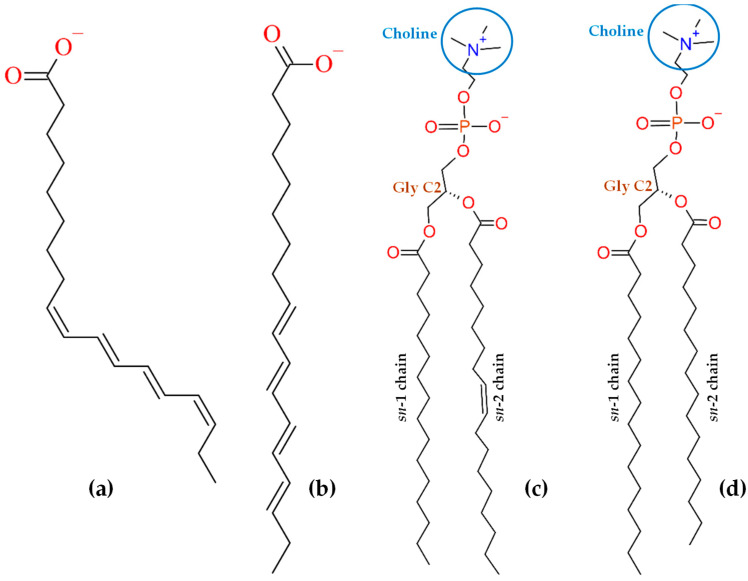
Structures of ionized cis-parinaric acid (*c*-PnA, **a**) and trans-parinaric acid (*t*-PnA, **b**), and phospholipids POPC (**c**) and DPPC (**d**), with indication of relevant atoms or groups of atoms.

Often, extrinsic fluorescent probes possess polar fluorophores, such as nitrobenzoxadiazole or rhodamine-based ones, which do not deeply penetrate lipid bilayers. Even if attached to the end of an alkyl or acyl chain, opposite to the head groups, these polar (and often bulky) fluorescent moieties tend to orient toward the water/lipid interface. Thus, they do not report on the hydrocarbon core where they were supposed to reside and are prone to inducing significant local perturbation of bilayer properties. Apolar fluorescent probes that insert lipid bilayers deeply include pyrene and pyrene-lipids, diphenylhexatriene and its trimethylamino derivative, and polyene probes *cis*- and *trans*-parinaric acids (*cis*, *trans*, *trans*, *cis*-9,11,13,15-octadecatetraenoic acid or *c*-PnA, and *all trans* -9,11,13,15-octadecatetraenoic acid or *t*-PnA, respectively). While the former two classes of probes have been studied in detail using atomistic MD simulations (see, e.g., Refs [10,11,12,13] and Refs [14,15,16,17], respectively), parinaric acids have so far been overlooked, the sole computational study consisting of a mean-field approach using Brownian dynamics simulation of *t*-PnA [18]. While this simple study reveals useful insights regarding chain orientation distributions, it does not provide a comprehensive description of the probe in all aspects of its behavior inside membranes. To fill this void, we present here MD simulations of *t*-PnA and its isomer *c*-PnA in fluid and gel bilayers of monounsaturated 1-palmitoyl-2-oleoyl-*sn*-glycero-3-phosphocholine (POPC) and saturated 1,2-dipalmitoyl-*sn*-glycero-3-phosphocholine (DPPC), respectively. The structures of both probes and host lipids are shown in Figure 1.

*c*-PnA is a naturally occurring fatty acid, found in the seeds of the Makita tree (*Atuna excelsa*) from southeast Asia and southwestern Pacific islands, among other sources, while *t*-PnA may be prepared by sunlight isomerization of *c*-PnA, catalyzed by iodine [19]. As shown in Figure 1, both isomers present four conjugated double bonds responsible for their absorption, with several discernible vibronic bands in the 270–320 nm wavelength range (with molar absorption coefficients of 8–9 × 10^4^ M^−1^cm^−1^) and broad emission with maximum near 410 nm in methanol [19]. While the fluorescence quantum yield of both probes in water is very low, and in polar protic solvents such as methanol it is still rather modest (0.015 ± 0.003; [19]), it increases markedly in less polar, non-protic solvents, and notably in lipid bilayers [20].

The spectroscopic properties of the membrane-inserted parinaric acids include a marked increase in fluorescence quantum yield, lifetime and anisotropy below the main transition temperature of one-component phospholipid systems [20]. Their fluorescence decays are complex both in solution and when inserted in membranes, and, in the latter medium, *t*-PnA exhibits a very long lifetime component in the gel phase (e.g., 35 ns in DPPC at 25 °C [21]). Time-resolved anisotropy is almost invariant in the solid ordered gel phase, unlike in fluid bilayers, where it tumbles in the low-nanosecond time scale to a lower residual value (see Section 2.4 below). Although these properties are mostly common to both isomers, *t*-PnA displays a greater sensitivity in quantum yield, lifetime and anisotropy changes to its environment. While both probes partition extensively to membranes from the water phase, *t*-PnA shows a marked—and virtually unique among common fluorescent probes—preference for the gel phase (as discussed in detail in Section 2.5). Because of this property, together with the above-mentioned microenvironment sensitivity, this probe in particular shows a peculiar ability to detect the presence of small amounts of the gel phase in mixed lipid bilayers, a feature which has been exploited in the determination of lipid mixture phase diagrams (e.g., [21,22,23,24,25,26,27]). Furthermore, PnA probes are useful FRET acceptors from protein tryptophan residues in protein/lipid or protein/ligand studies (e.g., [28,29]) and donors to nitrobenzoxadiazole probes for monitoring changes in lipid organization [30]. While most studies in the literature reporting usage of PnA probes date from the period from the late 1970s to the late 2000s (probably related to their recent reduced commercial availability, especially in the case of *t*-PnA), they remain useful probes of lipid order, as evidenced in a recent protocol [31].

In this paper, we carry out MD simulations to better understand the behavior of the two parinaric acid probes (and notably their similarities and differences) in both gel and fluid membranes. Two types of MD studies were carried out: all-atom simulations, to obtain insights on location, orientation and dynamics of the probes, as well as their effects on host lipid bilayer and interactions established with lipids, solvent and solution counterions; and complementary coarse-grained (CG) simulations, which aimed to determine the free energy profiles associated with the insertion of *c*-PnA and *t*-PnA into both lipid phases, to relate to their partition behavior.

## 2. Results and Discussion

**Figure 2:**
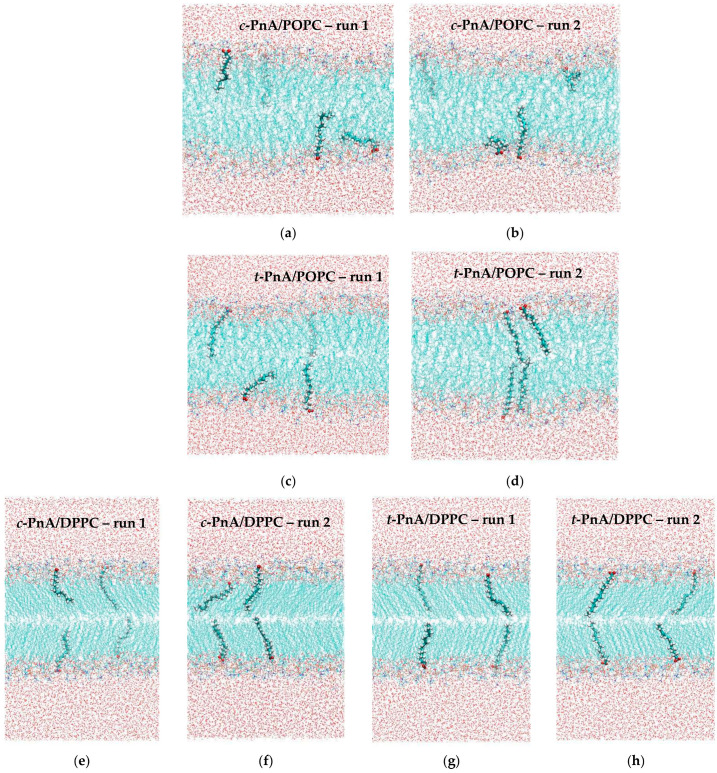
Final snapshots of the two runs of *c*-PnA in POPC (**a**,**b**), *t*-PnA in POPC (**c**,**d**), *c*-PnA in DPPC (**e**,**f**) and *t*-PnA in DPPC (**g**,**h**). For each lipid system, the four images are drawn on the same scale. However, for the sake of representation, the DPPC snapshots are drawn on a slightly reduced scale compared with the POPC ones.

As will be further described in Section 3.1, duplicate runs were carried out for each probe in the two lipid systems. Figure 2 shows the final snapshots obtained for each simulation.

Simple visual inspection of these snapshots reveals the most obvious differences between the two lipid bilayers (more ordered acyl chains in DPPC, with the characteristic tilt of the *L*_β_’ phase in evidence) and between the two probes (both orienting the carboxylate groups toward the water medium; *t*-PnA with its chain straighter and more aligned with those of the lipids compared with *c*-PnA). However, for a thorough comparison, extensive analysis of the different trajectories is required. As a preliminary step, we monitored the temporal variation of the carboxylate groups of each individual probe to verify that they are stable throughout the runs, mostly without systematic variations from the early stages of the simulations (Figure S1). For this reason, the properties described in the following subsections were calculated by averaging over the whole trajectories, except for the first 100 ns or 200 ns, in the POPC and DPPC simulations, respectively.

### 2.1. Bilayer Thickness and Area Per Lipid

**Figure 3:**
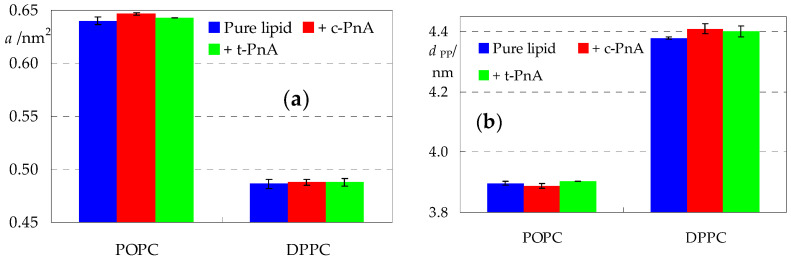
Average area per lipid molecule *a* (**a**) and bilayer thickness (evaluated as the distance between the average planes of the lipid P atoms in opposite leaflets, *d*_PP_; (**b**)) for the studied systems.

Aside from observation of discrete instant configurations, the most immediate analysis of a membrane MD simulation is the calculation of the average area per lipid *a*, which is simply computed as the instant simulation box area divided by the number of phospholipids in each leaflet and then averaged over all frames in the analysis time range. Values of 0.64 nm^2^ and 0.48 nm^2^ were obtained for pure POPC and DPPC, respectively (Figure 3a), in very good agreement with previous experimental (collected in [32,33]) and MD simulation (e.g., [34,35]) determinations. The incorporation of PnA probes increases *a* very slightly, mostly still within the statistical uncertainty associated with the value for pure lipid. It should be noted that *a* is still being calculated by dividing only by the number of phospholipids. If one considers, as approximation, that two single-chained PnA molecules contribute to the area of each leaflet to a similar (maybe slightly lower because of the much smaller polar head group) extent to that of one double-chained phospholipid, then an increase of up to 1% could be expected for a membrane with 100 lipid and 2 probe molecules per leaflet. This occurs in all cases, with the marginal exception of *c*-PnA in POPC (but even in this case, the further increase is not statistically significant).

As is well known, the bilayer thickness is highly negatively correlated with the area per lipid. Moreover, unlike *a*, its global value is not directly affected by inclusion of few amphiphilic molecules, such as parinaric acids, but may only be changed as a result of local effects of their insertion on the lipid molecules. We calculated the bilayer thickness as the distance between the average planes of the lipid P atoms in opposite leaflets (*d*_PP_; Figure 3b). In POPC, *c*-PnA and *t*-PnA decrease and increase *d*_PP_, respectively, which could nominally be interpreted as inducing disorder or order (respectively) to the host bilayer. However, the variations are not significant, as expected for this low probe content. In DPPC, both probes increase *d*_PP_ to values which could be marginally significant. At this stage of analysis, different possible explanations could be raised, such as ordering of the DPPC acyl chains (somewhat improbable, since they are already highly ordered in the gel phase) or a decrease in their tilt.

We also calculated deuterium order parameter profiles, which are depicted in Figure S2. The curve obtained for POPC agrees with both experimental [36] and computational [37] results. While no experimental profiles are available for gel phase DPPC, our profile agrees with that obtained from simulation by Curdová et al. [11]. The incorporation of probes produces minor effects, consistent with those observed for *a* and *d*_PP_. Still, it should be noted that the effects on all these bulk properties are very slight, confirming that parinaric acids are very mild probes in this respect, as hypothesized since the earliest published membrane studies using them [19].

### 2.2. Probe Location and Orientation

**Figure 4:**
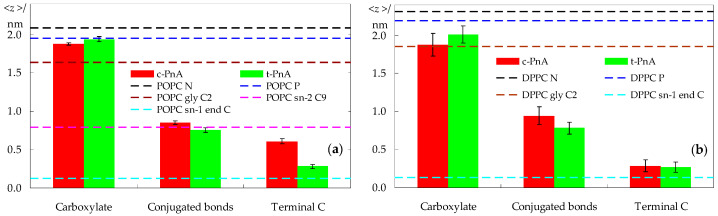
Average transverse distances to the bilayer center of mass for three probe groups (in columns) and, as reference, for different lipid atoms in probe-free systems, calculated for the POPC (**a**) and DPPC (**b**) simulations. Error bars reflect standard errors for the 95% confidence level.

We now turn from lipid to probe properties. The average transverse locations of different regions of the probes are shown in Figure 4. In POPC bilayers, the carboxylate group of the parinaric acids is anchored close to the lipid head group, at a location indistinguishable from that of the lipid P atom for *t*-PnA, and at a slightly deeper one for *c*-PnA. As seen in Figure 4a, the *t*-PnA molecules virtually span the entire membrane leaflet, almost reaching the depth of the POPC *sn*-1 terminal carbon atom (and actually matching that of the *sn*-2 terminal carbon atom; not shown). At variance, the *cis* configuration of the C9–C10 and C15–C16 bonds in *c*-PnA impedes the chain from reaching the innermost region of the fluid POPC bilayer. Still, the fluorophores of the two probes are located at a similar depth, near that of the C9–C10 double bond in the *sn*-2 chain of POPC.

Because of the considerably less dynamic nature of the DPPC gel phase bilayer, different individual probe molecules tend to display distinct behaviors. This translates into a larger uncertainty in their averaged locations, expressed in Figure 4b as longer error bars. While this implies that it is more difficult to arrive at definite conclusions, the observed trends seem to indicate that the carboxylate of *t*-PnA has a slightly shallower location than that of *c*-PnA. In any case, because of the increased bilayer thickness of DPPC, the carboxylates of both probes are positioned more deeply, in relative terms, than in POPC. Conversely, the opposite ends of the two probes have similarly deep locations. For *t*-PnA in DPPC, both below and above the main transition temperature, a deep location was inferred experimentally from fluorescence quenching experiments using fatty acids bearing a quencher doxyl spin label attached at different positions along the chain [38]. The more efficient quenching observed there by 16-doxylstearic acid than by 5-doxylstearic acid is fully compatible with our simulations.

**Figure 5:**
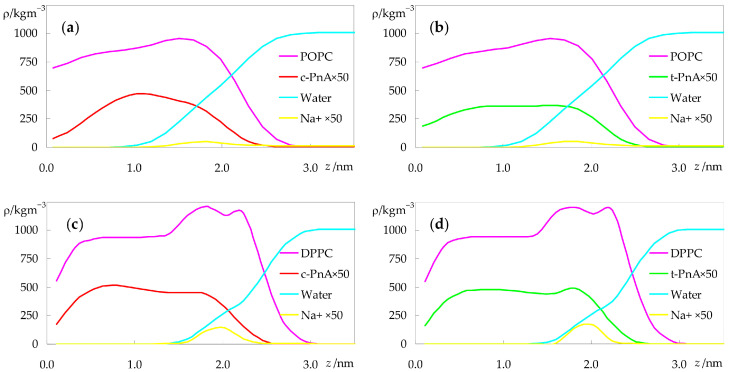
Mass density profiles of the different species in the simulations with *c*-PnA (**a**,**c**) or *t*-PnA (**b**,**d**), in POPC (**a**,**b**) or DPPC (**c**,**d**).

Another way to look at the transverse location of the probes is to examine the mass density profiles across the bilayer normal, shown in Figure 5.

The differences between the distributions of *c*-PnA and *t*-PnA in POPC are clear, with the latter reaching deeper into the bilayer, similarly to the host lipid (Figure 5b), whereas the density profile of the former virtually vanishes near the center of the bilayer (Figure 5a). However, the transverse distributions of the two probes are more similar in DPPC (Figure 5c,d), as already apparent in the atomic locations of Figure 4b. The increased order of the gel phase bilayer seems to force *c*-PnA to adopt a conformation closer to that of *t*-PnA.

**Figure 6:**
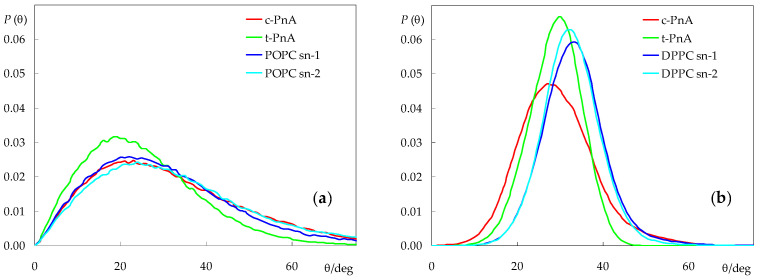
Long axis (defined as the vector from the terminal to the first C atom) tilt angle distributions of the parinaric acid and lipid acyl chains in POPC (**a**) and DPPC (**b**). Lipid distributions refer to probe-free simulations.

This can be confirmed by inspecting the long axis tilt angle distributions, both for the fatty acid probes and the host lipid acyl chains (Figure 6). As expected, the latter are wide for the fluid POPC bilayer (average values 30.0° (*sn*-1) and 32.5° (*sn*-2)) and more narrow for DPPC (average values 32.7° (*sn*-1) and 32.1° (*sn*-2), in excellent agreement with the experimental value of (32.0 ± 0.5)°, measured at 19 °C [39]). In POPC, the *c*-PnA chain adopts an orientation distribution very similar to those of the phospholipids (average value 30.9°), indicating an easy fitting of this probe within the hydrocarbon region of the membrane. In comparison, the *t*-PnA chain distribution is displaced to shorter angles (average value 24.6°), suggesting that this probe is not as easily accommodated in fluid bilayers as *c*-PnA and that its insertion may induce a slight ordering of the host lipid acyl chains, which is actually compatible with the very small increase in bilayer thickness observed for the *t*-PnA/POPC system (Figure 3b).

For DPPC, both probes display the orientation distributions which are displaced to lower angles compared to those of the phospholipid. *t*-PnA has a narrow distribution (even more so than those of DPPC), with a peak at 30° and an average of 28.4°. In contrast, *c*-PnA has a clearly wider distribution, with a slightly higher average (28.9°) but lower peak (27°). For *t*-PnA, our distributions closely resemble those experimentally obtained in the gel phase [40] and calculated from Brownian simulation in both phases [18] by Fernandes, Castanho and co-workers, with small differences (compared to the literature data, our distributions are very slightly displaced toward higher values) attributable to the experimental system (Langmuir–Blodgett multilayers), the small deviation between the electronic transition moment and the molecular axis [41], and differences in simulation methodologies.

**Figure 7:**
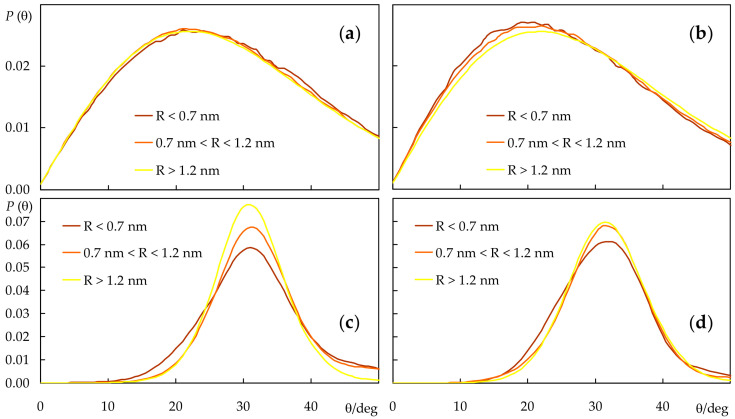
*sn*-1 acyl chain long axis (defined as the vector from the terminal to the first C atom) distributions of POPC (**a**,**b**) and DPPC (**c**,**d**), in the presence of *c*-PnA (**a**,**c**) or *t*-PnA (**b**,**d**). Each curve concerns a different range of distances *R* between the centers of mass of the lipid and the nearest probe.

Although, for simplicity, the lipid chain tilt distributions shown in Figure 6 only refer to probe-free simulations, they were also calculated for the systems containing probes. The overall effects are small, with the largest average deviation being recorded for the *sn*-2 chain in the presence of *t*-PnA (average tilt reduced by 0.5°) in POPC and for both chains in the presence of *c*-PnA in DPPC (average tilts reduced by 0.7° and 0.6° for the *sn*-1 and *sn*-2 chains, respectively). This is expected because of the relatively low probe concentration in the bilayer, which implies that most lipid acyl chains do not come into contact with probe molecules during the simulations. Still, these reductions in overall acyl chain tilt correlate with increases in bilayer thickness for the same systems (Figure 3b). For a more detailed picture, we analyzed the lipid *sn*-1 tilt distributions in the presence of probes, taking into account the distance *R* of each lipid to the nearest probe molecule in the same leaflet, for each simulation frame. We binned the tilts into three *R* categories: those of molecules with center of mass closest to that of the nearest probe (*R* < 0.7 nm), those of molecules with no probes in their immediate vicinity (*R* > 1.2 nm) and those in an intermediate situation (0.7 nm < *R* < 1.2 nm), as shown in Figure 7. From this analysis, it is confirmed that, in POPC fluid bilayers, even the lipid molecules closest to the probes are not significantly perturbed. For *t*-PnA, a small extent of induced ordering is apparent (28.4° of average tilt for *R* < 0.7 nm compared to 30.0° overall), in agreement with the lower average tilt of this probe. Conversely, for *c*-PnA, the lipid tilts are virtually unaffected (30.0° of average tilt for *R* < 0.7 nm compared to 30.1° overall), which agrees with the essentially identical chain tilts for this probe and POPC. More significant effects are observed for DPPC bilayers. Although the peak and average tilt angles are not affected by the presence of either *c*-PnA or *t*-PnA (differences are ≤ 1° in all cases), a widening of the *sn*-1 chain distributions is apparent for lipids closest to the probe molecules. This effect is more marked with *c*-PnA. For this probe, the distribution variance increases from 33.5 deg^2^ for *R* > 1.2 nm to 64.4 deg^2^ for 0.7 nm < *R* < 1.2 nm and 85.1 deg^2^ for *R* < 0.7 nm. In comparison, the corresponding variation for *t*-PnA ranges from 37.3 deg^2^ to 49.6 deg^2^ to 51.4 deg^2^. This means that the standard deviation of the distribution increases by 59% from DPPC molecules with no *c*-PnA in their vicinity to those with *c*-PnA neighbors, while the corresponding increase factor for *t*-PnA is only 17%. These variations reflect the wider distribution of *c*-PnA’s own tilt angles compared to *t*-PnA, which induce a similarly wider range of DPPC acyl chain tilts in nearby lipid molecules.

### 2.3. Interactions between Probes and Lipids or Solvent

**Figure 8:**
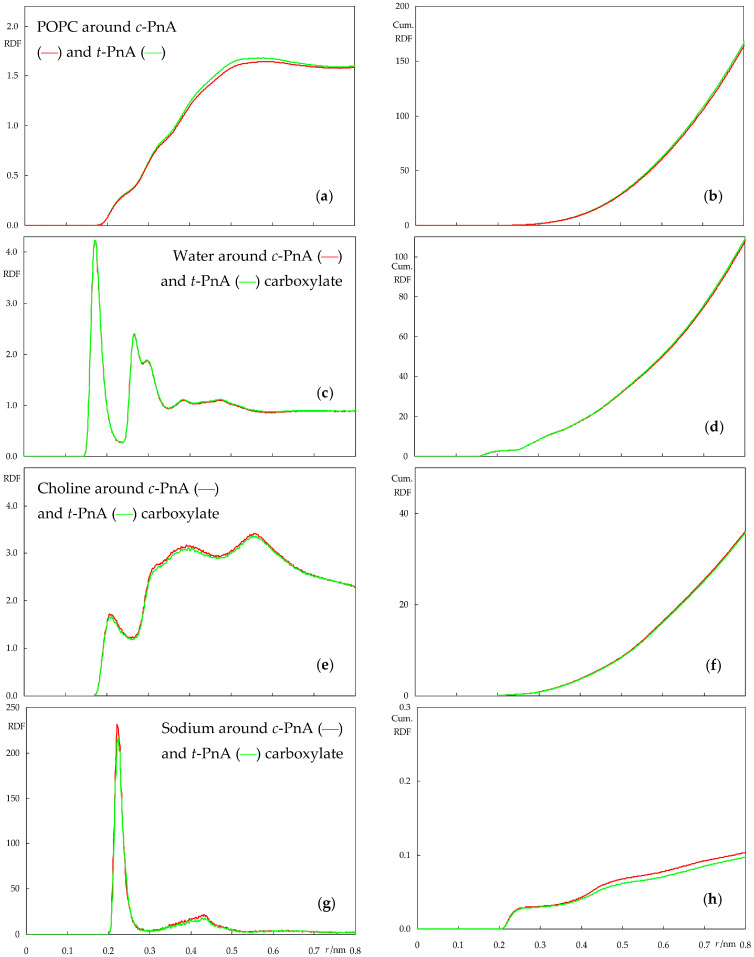
Atom–atom radial distribution density functions (RDFs, left plots) and their cumulative counterparts (cumulative RDFs, right plots) of lipid around PnA (**a**,**b**) and water (**c**,**d**), lipid choline (**e**,**f**) and sodium ion (**g**,**h**) around PnA carboxylate, calculated for the POPC simulations. Red and green curves refer to simulations with *c*-PnA and *t*-PnA, respectively.

**Figure 9:**
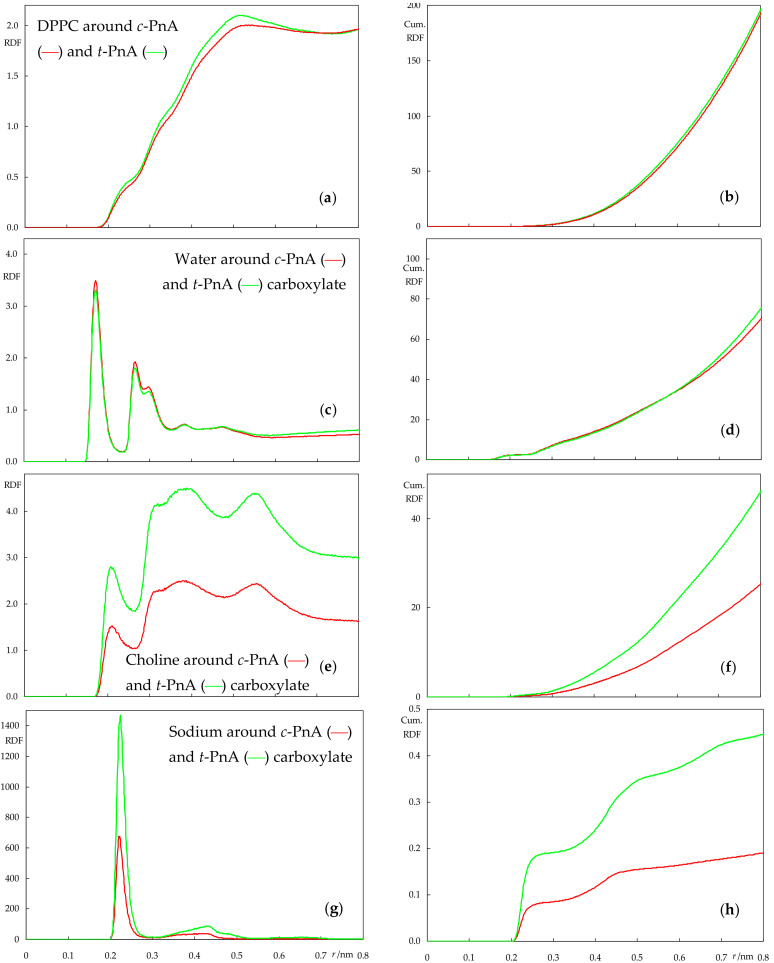
Atom–atom radial distribution density functions (RDFs, left plots) and their cumulative counterparts (cumulative RDFs, right plots) of lipid around PnA (**a**,**b**) and water (**c**,**d**), lipid choline (**e**,**f**) and sodium ion (**g**,**h**) around PnA carboxylate, calculated for the DPPC simulations. Red and green curves refer to simulations with *c*-PnA and *t*-PnA, respectively.

The location and orientation of bilayer-inserted *c*-PnA and *t*-PnA are closely linked with the interactions that probe molecules establish with both the surrounding lipids and solvent. To obtain insight on these interactions and how they may differ for the two probes, we calculated the atom–atom radial distribution functions (RDFs) of several system components around them in POPC (Figure 8) and DPPC (Figure 9): phospholipids around probes (Figure 8a,b and Figure 9a,b), and water molecules, lipid choline groups and sodium ions specifically around the carboxylate group of *c*-PnA and *t*-PnA (Figure 8c,d and Figure 9c,d; Figure 8e,f and Figure 9e,f; and Figure 8g,h and Figure 9g,h, respectively). RDFs were calculated using the default GROMACS gmx rdf normalization option, *i.e.* normalization for bin volume and density of selection groups. Because we are interested in the immediate vicinity of the fluorescent probe reference group, we restrict our discussion to distances ≤ 0.8 nm (extended RDFs, spanning ≥ 3.5 nm, are shown in Figures S3 and S4).

From Figure 8, it is clear that there is very little difference between the RDFs of two probes when inserted in POPC. The most significant difference is probably that of the RDF of POPC molecules around the probes, which has very slightly higher values for *t*-PnA. This probably stems from the straighter molecular shape of *t*-PnA, which is conducive to tighter lipid packing around it in comparison to *c*-PnA. Still, the effect is very minor in this fluid and disordered lipid system. Other than that, in our simulation, sodium appears to be more enriched around the carboxylate of *c*-PnA than around that of *t*-PnA, but the difference is small, probably within the higher uncertainty of this calculation; note that there are only four sodium ions in each simulation box, meaning that at any given time, they are mostly associated with lipid phosphates rather than with the outnumbered probe carboxylates, as evident from the cumulative numbers in Figure 8h, which are clearly lower than the maximal possible value of 4.

The RDFs in the DPPC system, shown in Figure 9, permit a more visible differentiation between the two probes. For a start, while the RDFs of lipid around both probes are higher than in POPC (because of the tighter packing in the gel phase DPPC bilayer compared to the fluid POPC), the difference between the two is now clearer (Figure 9a), as the almost linear fatty chain of *t*-PnA can be more easily accommodated within the rigid gel compared to the more twisted one of *c*-PnA. Elsewhere, *t*-PnA establishes considerably more frequent interaction with the positively charged DPPC choline groups and solvent sodium ions compared to *c*-PnA.

The two cis double bonds of *c*-PnA lead to a shorter end-to-end length when compared to *t*-PnA, as seen in Figure 4, potentially reducing the favorable hydrophobic interactions with lipid acyl chains. To counteract this, the molecule adopts a deeper carboxylate position, as well as a wider orientation distribution of its fatty chain long axis, with a peak at lower angles (Figure 6b). In turn, the deeper position, despite not affecting hydrogen bonding from water in our simulations (which is actually very slightly higher than for *t*-PnA, Figure 9c), leads to decreased favorable electrostatic interactions with lipid head groups and counterions. On the other hand, the wider tilt angle distribution of *c*-PnA in DPPC induces larger and longer ranged perturbation on the surrounding lipid acyl chains compared to *t*-PnA (Figure 7c,d), which possibly also contributes to the above-mentioned more evident difference in the RDFs of lipid around the probes in this system (Figure 9a).

### 2.4. Rotational and Translational Dynamics

As mentioned in the Introduction section, one property of particular interest in the behavior of *c*-PnA and *t*-PnA is its time-dependent fluorescence anisotropy, *r*(*t*). This observable is a measure of the polarization of the emission, which decays over time because of rotation experienced by the fluorophore. In an MD trajectory, rotation can be assessed by calculating rotational autocorrelation functions (ACFs):

(1) $$C(t)=\langle P_{2}(\cos\theta (\xi )\rangle$$

In this equation, θ(ξ) is the angle between the fluorophore long axis (the vector between C9 and C16 atoms of PnA) at times ξ and *t* + ξ, and *P*_2_ is the second Legendre polynomial. Averaging is carried out over both ξ and the eight simulated molecules of each species. Because the electronic transition dipole vector of linear conjugated polyenes, such as PnA, is roughly oriented along the direction of the C9–C16 vector [41], *C*(*t*) is expected to be approximately proportional to *r*(*t*) [42,43], the proportionality constant being *r*_0_, the fundamental anisotropy, which is very close to the maximum value of 0.4 for the PnA probes (see the discussion below).

**Figure 10:**
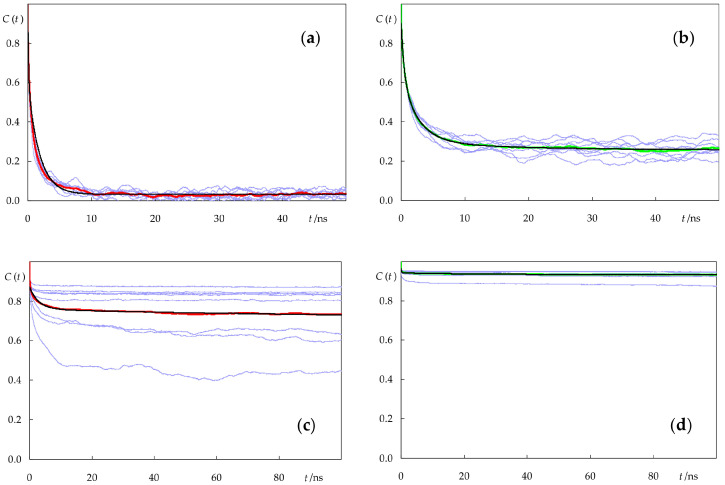
Rotational ACFs *C*(*t*) of *c*-PnA (**a**,**c**) and *t*-PnA (**b**,**d**) in POPC (**a**,**b**) and DPPC (**c**,**d**). Each thin blue line represents the *C*(*t*) of an individual fluorophore. The red and green lines (for *c*-PnA and *t*-PnA, respectively) represent the average over the eight simulated molecules of each probe in a given system. The black lines are fits to multiexponential functions (Equation (2); see Table 1 for best fit parameters).

Figure 10 shows the rotational ACFs of the eight individual molecules for each lipid/probe combination, as well as their averages. It is clear that different molecules of both *c*-PnA and *t*-PnA have similar rotational dynamics when inserted in the fluid POPC bilayer. This is not the case in DPPC, especially for *c*-PnA. The very slow dynamics of the gel phase implies that each molecule undergoes rotation with distinct kinetics, and in this case, averaging over a large number of molecules is required for an accurate calculation. While the relatively small number of molecules simulated here still implies relatively high uncertainty, we are reasonably confident that their average behavior may be compared with the experimental results and discussed, at least qualitatively.

The average curves were analyzed with a triexponential function with a finite residual term

(2) $$C(t)=a_{1}\exp(-t/\Phi_{1})+a_{2}\exp(-t/\Phi_{2})+a_{3}\exp(-t/\Phi_{3})+a_{\infty }$$

The best fit curves are also shown in Figure 10, while the respective parameters are summarized in Table 1.

In all systems, rotational reorientation occurs in the nanosecond timescale. For *c*-PnA, two exponential terms with similar amplitudes (one in the sub ns and the other in the ns time range) were recovered in POPC, while for DPPC, the exponential terms were in the ns and 100 ns time ranges. In both cases, there was no improvement in the goodness of fit when allowing a third exponential. Such an additional term was, however, recovered in the *t*-PnA fits. In any case, the most prominent feature of these functions is the residual term, *a*_∞_. The existence of such a term is also observed in the anisotropy decay analysis of membrane-inserted fluorophores, and it is commonly interpreted as arising from “wobbling-in-cone”-type hindered rotation [16,42,44]. In POPC, *a*_∞_ is relatively low, as expected from the fluid disordered nature of the bilayer. However, in DPPC, *a*_∞_ is very high because of the considerable hindrance to rotational motions in this solid ordered membrane. In both bilayers, rotation of the fluorophore of *t*-PnA is more impeded than that of *c*-PnA, to the extent that *t*-PnA is almost rotationally frozen in DPPC, except for a short amplitude tumbling.

These results may be compared with experimental measurements of time-resolved anisotropy. Mateo et al. [21] measured a zero-time anisotropy value of 0.39 for *t*-PnA in DPPC, very close to the maximal theoretical value of 2/5, and a residual component *r*_∞_ of 0.35 at 30 °C, confirming the limited extent of rotation of this probe in the gel phase. In another study [45], the same group measured a zero-time anisotropy of 0.29 for *t*-PnA in POPC at 30 °C (the smaller value probably denoting the existence of very fast depolarization, beyond the time scale available for the experiment) and a residual value *r*_∞_ = 0.14. A rough comparison of these limiting anisotropies with our *a* _∞_ values may be performed by multiplying the latter by 2/5, yielding estimates of 0.37 and 0.10 for DPPC and POPC, respectively, in fair agreement with the experimental values. Our rotational parameters for *t*-PnA also agree very well with the Brownian simulation estimates of Fernandes et al. [18]. For *c*-PnA, *r*_∞_ values of 0.04–0.07 and 0.30–0.34 have been reported for fluid and gel phase lipids, respectively [46]. A simple multiplication of our recovered *a*_∞_ values by 2/5 would yield estimates of 0.01 and 0.28, respectively, somewhat below those values. However, it should be noted that the gel phase value, in particular, is subject to increased uncertainty, as commented above, and the fact that a long correlation time of 120 ns (with amplitude *a*_2_ = 0.050) is recovered for this system may justify the small difference, since very long decay times are numerically strongly correlated with limiting asymptotic values. In any case, our calculations seem to confirm the experimental observations of larger rotational hindrance of the fluorophore of *t*-PnA compared to that of *c*-PnA in the same systems. The lower residual anisotropy of *c*-PnA has been attributed to its non-linear shape, namely an extra depolarizing motion consisting of a rotation of the chain around its axis in addition to the other motions that occur [47]. Although the conjugated bonds of *t*-PnA and *c*-PnA have slightly different locations in both lipid systems (Figure 4), the difference is very small and probably could not alone justify this large discrepancy. In this regard, the better alignment of the fatty acid chain of *t*-PnA with the lipid acyl chains in DPPC, the related tighter lipid packing around *t*-PnA in both lipid systems, and possibly also the increased interactions of *t*-PnA with lipid headgroups could also contribute to the observed differences in the behavior of the two probes.

We also addressed the translational diffusion of both probes in the bilayer plane in comparison to the host lipid in each case. For this purpose, we first calculated the two-dimensional mean squared displacements (MSD), defined by

(3) $$\mathrm{MSD}(t)=\langle {\left\|{\vec{r}}_{i}(t+t_{0})-{\vec{r}}_{i}(t_{0})\right\|}^{2}\rangle$$

where ${\vec{r}}_{i}$ is the (*x*, *y*) position of the center of mass of molecule *i* of a given species, and the averaging is carried out over all molecules of this kind and time origins *t*_0_ using trajectories with fixed center of mass of the monolayer where the solute is located to eliminate noise due to fluctuations in the center of mass of each bilayer leaflet.

In turn, MSD can be used to estimate the lateral diffusion coefficient *D_lat_* using the Einstein equation.

(4) $$D_{lat}=\frac{1}{4}\underset{t\to \infty }{\lim}\frac{d\mathrm{MSD}(t)}{dt}$$

Figure S5 displays the time variations of MSD for probes and host lipids in each system. For the fluid phase systems, using the data of Figure S5a,b, one could estimate *D_lat_* values of (5.5 ± 1.1) × 10^−8^ cm^2^s^−1^, (11 ± 3) × 10^−8^ cm^2^s^−1^ and (16 ± 1) × 10^−8^ cm^2^s^−1^ for POPC, *c*-PnA and *t*-PnA, respectively. It must be stressed that the significance of MSD plots and accurate calculation of lateral diffusion in membranes remains, to a great extent, a controversial problem. It depends largely on the available time window [16,48]. Sampling problems are more important in lateral diffusion than in some other properties because it involves large-scale motions of whole molecules rather than limited range/segmental motions (such as those involved in lipid acyl chains or probe long axis orientation). For relatively short times, lipid diffusion (as perceived by MSD variation) is mainly due to conformational changes of the hydrocarbon chains rather than diffusion of the entire molecule [48], and therefore, its meaning and its relationship with experimental observables are somewhat questionable. In this context, the value obtained for POPC is in good agreement with the pulsed field gradient NMR data of Filippov et al. [49], which we estimate as 7.8 × 10^−8^ cm^2^s^−1^ from Figure 6b of that reference. The faster diffusion observed for the probes compared to POPC may be justified by the fact that the former possess a single chain and do not establish strong interactions with the lipid head groups in this lipid system. The slower diffusion of *c*-PnA compared to *t*-PnA may be tentatively linked to the kinks in the chain of the former that result from its *cis* double bonds, which may cause slight hindrance in its lateral translational motion.

Turning to DPPC, we observe that diffusion in the gel phase occurs much more slowly than in the fluid phase (see Figure S5c,d, notably their much shorter ordinate scales compared to those of Figure S5a,b), as is well known. Based on our MSD plots, we could estimate *D_lat_* values of the order of ~10^−10^ cm^2^s^−1^ for the different species. Still, it must be stressed that a clear linear regime is not obtained for the MSD time variation, and even for DPPC (the species for which sampling is obviously more extensive), the slope seems to be still decreasing for longer times, in qualitative agreement with reports of *D_lat_* as low as ~10^−12^–10^−13^ cm^2^s^−1^ for DPPC supported on mica [50]. From the discussion in the previous paragraph, a poorer estimation of diffusion coefficients in the gel phase is not surprising, and for this reason, we do not attempt to calculate actual values and uncertainty intervals here. While the curves in Figure S5c,d reflect global averages, there is large variability among different probe molecules (not shown), implying that for the probes in particular, the estimated uncertainties are of the same order as the actual *D_lat_* values. Therefore, in this system, we refrain from a quantitative analysis and note only that, while lateral diffusion of *c*-PnA and DPPC seems to occur on a similar timescale (Figure S5c), that of *t*-PnA appears to be notably slower (Figure S5d). This could be related to the stronger interaction between its carboxylate and positively charged lipid choline groups (Figure 9e) and a lesser degree of local perturbation compared to *c*-PnA (Figure 7d vs. Figure 7c), which qualitatively agrees with the slower rotational dynamics characterized above. Both the slower fluorophore rotation and lateral translational diffusion of *t*-PnA indicate that motions of this probe are very limited in the gel phase, agreeing with the signature long intensity decay component observed experimentally (see Introduction).

### 2.5. Free Energy Profile of PnA across DPPC and POPC Bilayers and Its Relation to Probe Partition

The calculation of free energy profiles for the interaction of the probes with both POPC and DPPC lipid membranes was obtained from simulations at the CG level. The option of CG level simulations was motivated by the slow dynamics of the DPPC membranes, requiring larger simulation times for proper sampling and PMF convergence, and the reduced computational cost of CG simulations. The CG parametrization of both probes was obtained based on the previous Martini 2.2 parameters used for fatty acids, and further details may be found in supplemental information. The PMF profiles are used to obtain free energy barriers for the interaction of the probes with membranes, which will be compared to experimental results for the water/membrane partition of the probes.

**Figure 11:**
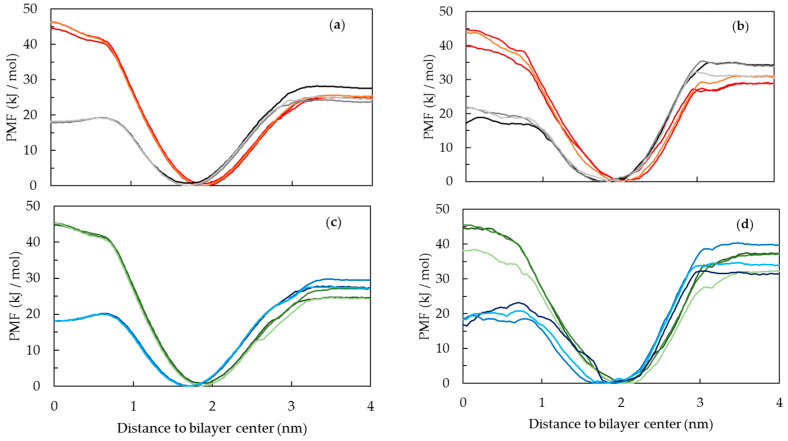
Potential of mean force (PMF) profiles across the lipid bilayers of *c*-PnA in POPC (**a**), *c*-PnA in DPPC (**b**), *t*-PnA in POPC (**c**) and *t*-PnA in DPPC (**d**). Data are presented with the following color code: ionized *c*-PnA (red), neutral *c*-PnA (black/gray), ionized *t*-PnA (green) and neutral *t*-PnA (blue). Curves with different shades of these colors correspond to profiles obtained from replicate sets of umbrella sampling simulations.

The PMF profiles for the interaction of the different PnA molecules with the lipid bilayers, shown in Figure 11, are consistent with the typical PMF profiles for the interaction of amphiphilic molecules with lipid bilayers. The PMF profiles show a free energy minimum at the lipid/water interface, a barrier for the translocation between bilayer leaflets and a plateau in the water phase [51]. All profiles show a very small or negligible energy barrier for the insertion of the probes from water to the membrane. On the other hand, the energetic penalty associated with the transfer of the probes from the equilibrium position in the membrane to the water phase is evident in all systems. This energetic penalty can be related to the desorption of the probes from the membrane to the water, and in absence of the energy barrier for the insertion process, it can also be related to the partition to the membranes.

A note should be given regarding sampling problems in the umbrella sampling simulations, namely in DPPC, which, if not considered, can be reflected in the calculated energy barriers. The PMF profiles spanning the whole bilayer thickness are shown in the Supporting Information, Figure S6. As reported previously [51], these problems originate when the pulling of the solute starts in the water, in this case at negative values of the reaction coordinate, as shown in Figure S6b,d. For this reason, the energy differences calculated in this work are calculated only with the information of the molecule pulled from the center of the bilayer to the water, i.e., from the positive region of the reaction coordinate.

**Figure 12:**
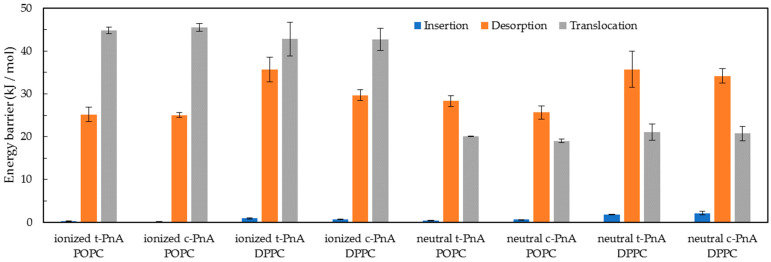
Energy barriers for the insertion (blue), desorption (orange) and translocation (gray) processes of ionized and neutral *t*-PnA and *c*-PnA in POPC and DPPC lipid bilayers.

From the PMF profiles, the energy differences for partition, insertion, desorption and translocation of the PnA molecules in the lipid bilayer were calculated, as shown in Figure 12. The translocation of fatty acids is a fast process due to the protonation of these molecules while passing through the hydrophobic core of lipid membranes [52]. For this reason, both deprotonated and protonated forms of the PnA were considered in these simulations. Differences in the PMF profiles of the two protonation states are mostly evident for the translocation energy barrier. The translocation energy barrier is higher for the deprotonated species due to the presence of the charged head group in the bilayer core. The shape of the translocation energy barrier is also different for the two species, presenting a small local minimum for the protonated species, evident for the POPC membranes, similar to that reported for other non-charged molecules, such as cholesterol [53]. Regarding the translocation of PnA in both lipid bilayers, the correspondent energy barrier for the protonated form is slightly higher in DPPC than in POPC, which seems reasonable regarding the higher packing and slower dynamics of the gel phase.

Regarding the partition to the lipid bilayers, as discussed below, the PMF profiles are in good agreement with experimental data [20] for the partition of PnA to liquid disordered and gel phases. The free energy difference between the water phase and the equilibrium position is larger for the DPPC than for the POPC bilayer, also in agreement with the larger partition of PnA probes for the gel phase (see below). Considering the different protonation species of the PnA, small differences are observed, although a larger free energy difference can be observed for the protonated species, in agreement with the higher hydrophobicity. This small difference can be interpreted, since the main contribution to the desorption energy barrier and to the partition of long chain amphiphiles comes mainly from the size of the chain, being less influenced by the head group of the amphiphilic molecule. However, considering the interfacial location of the PnA head group on the lipid membranes, the deprotonated form should be dominant and more relevant at the equilibrium position. Regarding the partition of *c*-PnA and *t*-PnA for the lipid bilayers, the energy difference for partition of *t*-PnA to DPPC is also higher than that of *c*-PnA, which is also plausible regarding the better packing of *t*-PnA in the gel phase DPPC.

We can use the barrier heights of Figure 12 to estimate the partition coefficients using *K*_p_ = exp(−ΔG(w→l)/*RT*), where ΔG(w→l) is the free energy for solute transfer from the water to the equilibrium location in the lipid bilayer, equal to the difference between the desorption and the insertion energy barriers. These *K*_p_ estimates may, in turn, be compared to experimental measurements (Table 2). It should be pointed out that an absolute comparison is hampered because of the lack of a clear direct correspondence between the experimental and the calculated values, in part because the monounsaturated lipid in the fluid phase is not the same in both sets of data (POPC in the simulation, 1-palmitoyl-2-docosahexaenoyl-*sn*-glycero-3-phosphocholine in the experiment), but mostly due to different underlying reference states.

Still, relative comparisons are possible using the PMFs of the ionized form, the one expected to predominate for COO positions in the water medium and the interfacial regions of the bilayer, up to the equilibrium position. For example, both experimental and computational sets of data indicate that: (i) *c*-PnA and *t*-PnA have similar values of *K*_p_ from water to the fluid phase; (ii) the partition coefficient of *t*-PnA to the gel phase is significantly higher than that of the same probe to the fluid; (iii) *t*-PnA has a much higher *K*_p_ from water to the gel phase (by a full order of magnitude) compared to *c*-PnA. The only comparison that does not hold well concerns the difference in *K*_p_ values of *c*-PnA between the two phases (less than two-fold lower for the gel in the experiments, and approximately four-fold higher for the gel in the simulation), but even in this case, there is no gross disagreement. On the whole, MD simulations reproduce the main features of the partition behavior of the two probes between water and both solid ordered and fluid disordered phases.

## 3. Methods

### 3.1. All-Atom MD Simulation

The all-atom MD simulations and analyses were carried out with GROMACS version 2019 [54]. The visualization of the trajectories was performed with Visual Molecular Dynamics (VMD) v.1.9.3 [55]. In these simulations, the CHARMM36 force field was employed for the lipid molecules [56], together with the updated version of the original Beglov and Roux parameters [57] for the ions [58], the CGENFF v4.4 for the parinaric acid molecules [59] and the modified version of the TIP3P model [60,61] to be used with CHARMM force field for the water molecules [62]. Even though the TIP3P water parameterization has been questioned in the literature [63], validation of the CHARMM36 lipid force field was carried out with this water model, and the resulting bilayer properties reproduce closely the available experimental data [56]. The parametrizations of *t*-PnA and *c*-PnA were performed using the Ligand Reader & Modeler tool [64] on the CHARMM-GUI platform [65]. The parinaric acids were parametrized in their anionic ionization state, since their predicted aqueous p*K*_a_ in water solution lies in the 4.8–5.0 range obtained from the Natural Products Magnetic Resonance Database, NP-MRD (Figure 1) [66]. In both cases, the parametrization procedure produced topologies with charge, bond and angle parameters with penalty scores lower than 10, meaning that the parameters attributed by analogy were fair, and no additional modifications were required. For the dihedral parameters, two of them were attributed a penalty score between 10 and 50, requiring some additional validation in both parinaric acids. For that purpose, in order to validate the full topology of each parinaric acid, production simulations of 12 ns were run after minimization, NVT and NPT equilibration steps. For the analysis, the first 2 ns of the simulations were excluded. For both parinaric acids, the averaged bond distance, angles and improper dihedral obtained from the simulation agreed with the parameters from the attributed topology (not shown). The pure membrane systems were built using the Membrane Builder tool from CHARMM-GUI [67]. For the POPC pure membrane system, a total of 200 lipids and 45 water molecules/lipid were added. The DPPC pure membrane system was built with the same number of lipids, and 50 water molecules/lipid were added. Therefore, fully hydrated phospholipid bilayers were simulated in both cases. The membrane systems were equilibrated with the CHARMM-GUI protocol, which included a minimization step and several small NVT and NPT equilibrations steps with position restraints, which were gradually alleviated until the restraint-free production run. This helps the membrane to equilibrate properly [68]. The production runs for the POPC and DPPC membranes were extended up to 200 ns and 2 µs, respectively. Several properties (e.g., area/lipid, deuterium order parameters, bilayer thickness and long axis tilt angle) were well reproduced compared to the literature experimental results, attesting the correct equilibration of the membranes (see Section 2.1 and Section 2.2). All the topology files obtained were converted to be compatible with the GROMACS software by the CHARMM-GUI platform [68].

Four parinaric acids were positioned inside the previously equilibrated 200-lipid membranes, two in each leaflet. The systems were subsequently neutralized with sodium ions using the GROMACS genion tool. Every system was minimized, followed by NVT and NPT 100 ps equilibration runs with a 1 fs integration step before the production run, eliminating bad contacts. The production runs for the systems with POPC and DPPC membranes were carried out for 500 ns and 1 µs, respectively. Two replicates were run for each system, changing the initial position of the parinaric acid, and with the initial velocities of the particles randomly attributed.

The production runs, for all the all-atom simulations performed in this work, were performed in NPT conditions. An integration step of 2 fs was employed. Periodic boundary conditions were applied. The electrostatics interactions were modeled with particle-mesh Ewald (PME) method [69] with a 1.2 nm cut-off. A force-based switch function within the 1.0–1.2 nm range was applied for the Van deer Waals interactions cut-off. The parinaric acids together with the membrane lipids, and the water molecules together with sodium ions, were coupled independently with temperature baths at 298.15 K using the Nose–Hoover algorithm [70,71] and with a time constant for coupling (tau-t) of 1 ps. The pressure was maintained constant at 1 bar with a barostat employing the Parrinello–Rahman algorithm [72] with a semi-isotropic scheme, a coupling constant of 5 ps and a compressibility of 4.5 × 10^−5^ bar ^−1^. Constraints in the H-bonds were applied using the LINCS algorithm [73], and no dispersion corrections were used.

### 3.2. CG Simulation and Calculation of PMF Profiles

All CG simulations were carried out using the Martini 2.2 force field [74], running in GROMACS 2019 and GROMACS 2022 [54,75]. DPPC parameters, with bonded terms optimized for Martini, such that their sampled populations best fit the distributions obtained from a pseudo CG mapping generated from the all-atom CHARMM36 force field, were taken from Ref [76], where it was also shown that this CG model, unlike standard Martini, reproduces the main transition at 314 K. Parameterization of *t*-PnA and *c*-PnA for Martini 2.2 was carried out by adapting the parameters used for fatty acids. Further details are given in the Supplemental Information provided within this article, including the mapping scheme (Figure S7) and a comparison between bond length (Figure S8) and angle (Figure S9) distributions for the CG and atomistic mapped parameterizations of the different PnA molecules.

The starting structures, fully hydrated POPC and DPPC bilayers, were built with the insane.py script to set up the Martini bilayer systems [77]. Both bilayers were composed of 162 lipids and 2250 water beads, 225 of which were anti-freeze water beads. Simulations were carried out under a constant number of particles, pressure (1 bar) and temperature (298.15 K, NPT), and with periodic boundary conditions. Temperature and pressure controls were carried out using the V-rescale thermostat [78] and the Berendsen barostat [79], respectively. Semi-isotropic pressure coupling was used. Coulomb interactions were calculated using the reaction field method with a cut-off of 1.1 nm and a dielectric constant of 15. The Lennard-Jones interactions were cut off at 1.2 nm. Lipid bilayers were equilibrated using the following protocol. Unfavorable atomic contacts were removed by steepest descent energy minimization, followed by a 1 ns and 10 ns equilibration run and a 10 µs production run (using 2 fs, 10 fs and 20 fs integration time steps, respectively). The final configuration of the 10 µs simulation of each bilayer, shown in Figure S10, was used in the umbrella sampling simulations.

The PMF profiles were obtained by sampling the behavior of two PnA molecules in each of the POPC and DPPC bilayers. The reaction coordinate was defined as the distance from the COO^-^ bead of the PnA molecules to the center of mass (COM) of the lipid membranes, calculated with a cylinder pull geometry [51]. PnA molecules were pulled simultaneously, one molecule starting from the center of the membrane and the other in the water phase, at a pulling rate of 0.0005 nm ps^−1^ and a force constant of 500 kJ mol^−1^ nm^−2^. From this simulation, the initial snapshots were obtained, separated by 0.1 nm, for a reaction coordinate window from −4 nm to 4 nm for POPC and from −5 nm to 5 nm for DPPC. PMF profiles were obtained for the deprotonated and protonated states of both PnA molecules. The initial frames obtained for the deprotonated PnA were also used for the sampling simulations of the protonated PnA molecules.

For each umbrella window, sampling simulations were run for 200 ns using a harmonic umbrella potential with a 3000 kJ mol^−1^ nm^−2^ force constant applied to the COO^−^ bead of the PnA molecules. The set of pulling/sampling simulations was performed in triplicate.

The convergence of the PMF profiles was tested as described in [51]. From the convergence analysis, it was also considered that discarding the initial 100 ns of the sampling simulations led to a consistent and systematic protocol for all calculated PMFs. The final PMF profiles were calculated considering the last 100 ns of the sampling simulations.

For the visualization of structures and trajectories, VMD software (University of Illinois, Urbana-Champaign, IL, USA) was used [55].

## 4. Conclusions

In this work, we carried out an MD simulation study of fluorescent probes *c*-PnA and *t*-PnA, inserted in both the gel phase (DPPC) and fluid phase (POPC) bilayers. Our CG simulations allowed us to obtain free energy profiles, which reproduced the experimentally observed extensive partition of *t*-PnA for the solid ordered phase compared to *c*-PnA. On the other hand, both probes partitioned to fluid bilayers to a similar extent, as also verified experimentally. From the calculated free energy profiles, relatively low energy barriers were inferred for the translocation across the bilayers of both probes in the neutral form, in accordance with their well-known fast equilibration between bilayer leaflets.

We carried out long all-atom simulations, which were extensively analyzed to provide the clues for this and other known features in the behavior of the two probes, which only differed in the configuration of two double bonds (C9–C10 and C15–C16) along their fatty acid chains. We verified that host lipids are more tightly packed around the almost linear *t*-PnA molecule than around the more twisted *c*-PnA isomer. In POPC bilayers, this has little bearing on partition because the fluid phospholipid acyl chains accommodate well the tail of *c*-PnA, which actually shows an orientational distribution very close to that of the host lipid. However, in the thicker DPPC membrane, *c*-PnA is forced to adopt a slightly lower position for its carboxylate. Overall, this limits the favorable interactions between *c*-PnA carboxylates and lipid choline groups and cationic counterions compared to *t*-PnA, which, combined with the difference in lipid packing around the two probes, may explain the disparity in the partition coefficient of the two probes in this environment.

Experimentally, it was observed that *c*-PnA and *t*-PnA exhibited different rotational motion kinetics. Our calculated rotational autocorrelation functions for the fluorophore axis agree well with fluorescence anisotropy decays, namely the higher residual component for *t*-PnA, especially in DPPC. The linear shape of the *t*-PnA fluorophore implies a reduction in degrees of rotational freedom, which, conjugated with the tighter lipid packing, justifies the observed difference. Actually, both rotational and translational motions of *t*-PnA are highly impeded in DPPC, which is also in accordance with the long intensity decay component typical of this probe in gel phases.

Overall, our study confirms that *c*-PnA and *t*-PnA are highly valuable fluorescent membrane probes. Absorbance in the ultra-violet and propensity for oxidation, leading to bleaching, are the obvious drawbacks in microscopy studies, but they can be minimized, e.g., by using multiphotonic excitation [80]. *c*-PnA is a naturally occurring compound, with large relative availability. It is an excellent probe, especially for the fluid phase, where its perturbation of host lipid properties is minimal. While its partition to the gel is less extensive than that of *t*-PnA, it is still much higher than those of probes with extrinsic fluorophores, which are often excluded from solid ordered phases. On the other hand, *t*-PnA is simply a distinctive probe in this regard. While a considerable amount of experimental data are available for the two probes, the present study allows an unprecedentedly deep insight on atomic-scale properties, which help explain their behavior when inserted in lipid bilayers. This novel information may contribute to a timely reappraisal of these very useful probes of lipid order. While we chose for this study simple one-component lipid systems, based on representative phosphatidylcholines that exist in stable fluid or solid phases at room temperature (and for which experimental data are available for comparison), other host lipid compositions, with varying head groups and acyl chain lengths/saturation degrees, as well as containing cholesterol, may be explored in future studies.

## Figures and Tables

**Table 1:** Best fit parameters of Equation (2) to the calculated rotational ACFs.

Host Lipid	POPC		DPPC	
Probe	*c*-PnA	*t*-PnA	*c*-PnA	*t*-PnA
*a*_1_	0.42	0.27	0.11	0.02
Φ_1_/ns	0.10	0.46	2.9	0.16
*a*_2_	0.58	0.33	0.050	0.01
Φ_2_/ns	1.8	2.6	120	22
*a*_3_	-	0.047	-	0.01
Φ_3_/ns	-	15	-	850
*a*_∞_	0.032	0.26	0.71	0.92
*<*Φ*>*/ns *	1.1	1.7	5.2	2.3

* Calculated from integration of the best fit curve.

**Table 2:** Published experimental lipid/water partition coefficient values for *c*-PnA and *t*-PnA in both gel and fluid phases [20] and the estimates from the PMF profiles determined for the ionized PnAs in this work.

Lipid Phase	Fluid		Gel	
Probe	*c*-PnA	*t*-PnA	*c*-PnA	*t*-PnA
Experimental	9 × 10^5 a^	1.7 × 10^6 a^	5.3 × 10^5 b^	5 × 10^6 b^
Estimate from simulation	2.3 × 10^4^	2.4 × 10^4^	1.3 × 10^5^	1.2 × 10^6^

^a^ 1-palmitoyl-2-docosahexaenoyl-*sn*-glycero-3-phosphocholine at 22 °C. ^b^ DPPC at 22 °C.

## Data Availability

All force field topologies newly developed for this work are available as Supplementary Material. Other original data will be provided by the corresponding authors upon request.

## Supplementary Materials

The following supporting information can be downloaded at: https://www.mdpi.com/article/10.3390/molecules28052241/s1, All-atom simulations—Figure S1: Time dependence of the positions of the carboxylate group of c-PnA and t-PnA in POPC and DPPC; Figure S2: Deuterium order parameter profiles calculated for the phospholipid sn-1 acyl chains; Figures S3 and S4: Fully extended RDFs in POPC and DPPC systems, respectively; Figure S5: Time variation of lateral mean square displacements for the POPC and DPPC simulations with inserted c-PnA or t-PnA; Probe force field files for use with CHARMM36; Coarse-grained simulations—Free energy profile of PnA across DPPC and POPC bilayers (Figure S6); Topologies for protonated and unprotonated c-PnA and t-PnA; Mapping scheme (Figure S7); Comparison of bond length and angle distributions for the Martini and atomistic mapped parameterization of PnA molecules (Figures S8 and S9); Snapshots of POPC and DPPC membranes used in the calculation of PMF profiles (Figure S10). Reference [51] is cited in the supplementary materials.
