# Supplementary material for: Fluorescent Probes cis- and trans-Parinaric Acids in Fluid and Gel Lipid Bilayers: A Molecular Dynamics Study
Source: Molecules. 2023 Feb 28;28(5):2241. doi: 10.3390/molecules28052241 (PMC10005308; doi:10.3390/molecules28052241)
Supplement: Supplementary file 1 [file molecules-28-02241-s001.zip › Oliveira_et_al_Supplemental_Material.pdf]

Fluorescent probes *cis*- and *trans*-parinaric acids in fluid and gel lipid bilayers: a molecular dynamics study

Alexandre C. Oliveira [1,2,†], Hugo A. L. Filipe [1,3,*,†] and Luís M. S. Loura [1,4,5,*]

[1] Coimbra Chemistry Center, Institute of Molecular Sciences (CQC-IMS), University of Coimbra, 3004-535 Coimbra, Portugal
[2] Department of Chemistry, Faculty of Sciences and Technology, University of Coimbra, 3004-535 Coimbra, Portugal
[3] Polytechnic of Guarda, CPIRN-IPG—Center of Potential and Innovation of Natural Resources, 6300-559 Guarda, Portugal
[4] Faculty of Pharmacy, University of Coimbra, 3000-548 Coimbra, Portugal
[5] CNC—Center for Neuroscience and Cell Biology, University of Coimbra, 3004-535 Coimbra, Portugal
* Correspondence: hlfilipe@ipg.pt (H.A.L.F.); lloura@ff.uc.pt (L.M.S.L.)
† These authors contributed equally to this work.

Contents

Supplementary data from the all-atom simulations S2

Time dependence of the positions of the carboxylate group of *c*-PnA and *t*-PnA, in POPC and DPPC S2

sn-1 acyl chain order parameter profiles in the absence and in the presence of PnA S3

Fully extended radial distribution functions in the POPC simulations S4

Fully extended radial distribution functions in the DPPC simulations S5

Time variation of lateral mean square displacements for the POPC and DPPC simulations with inserted *c*-PnA or *t*-PnA S6

Free energy profile of PnA across DPPC and POPC bilayers S7

Coarse-grained parameters for PnA molecules S8

Comparison of bond length and angle distributions for the Martini and atomistic mapped parameterization of PnA molecules S11

Snapshots of POPC and DPPC membranes used in the calculation of PMF profiles S13

Reference S13

Appended files: all-atom topologies for *cis*- and *trans*-parinaric acid, for use with CHARMM36 lipid bilayers.

Supplementary data from the all-atom simulations

Time dependence of the positions of the carboxylate group of c-PnA and t-PnA, in POPC and DPPC

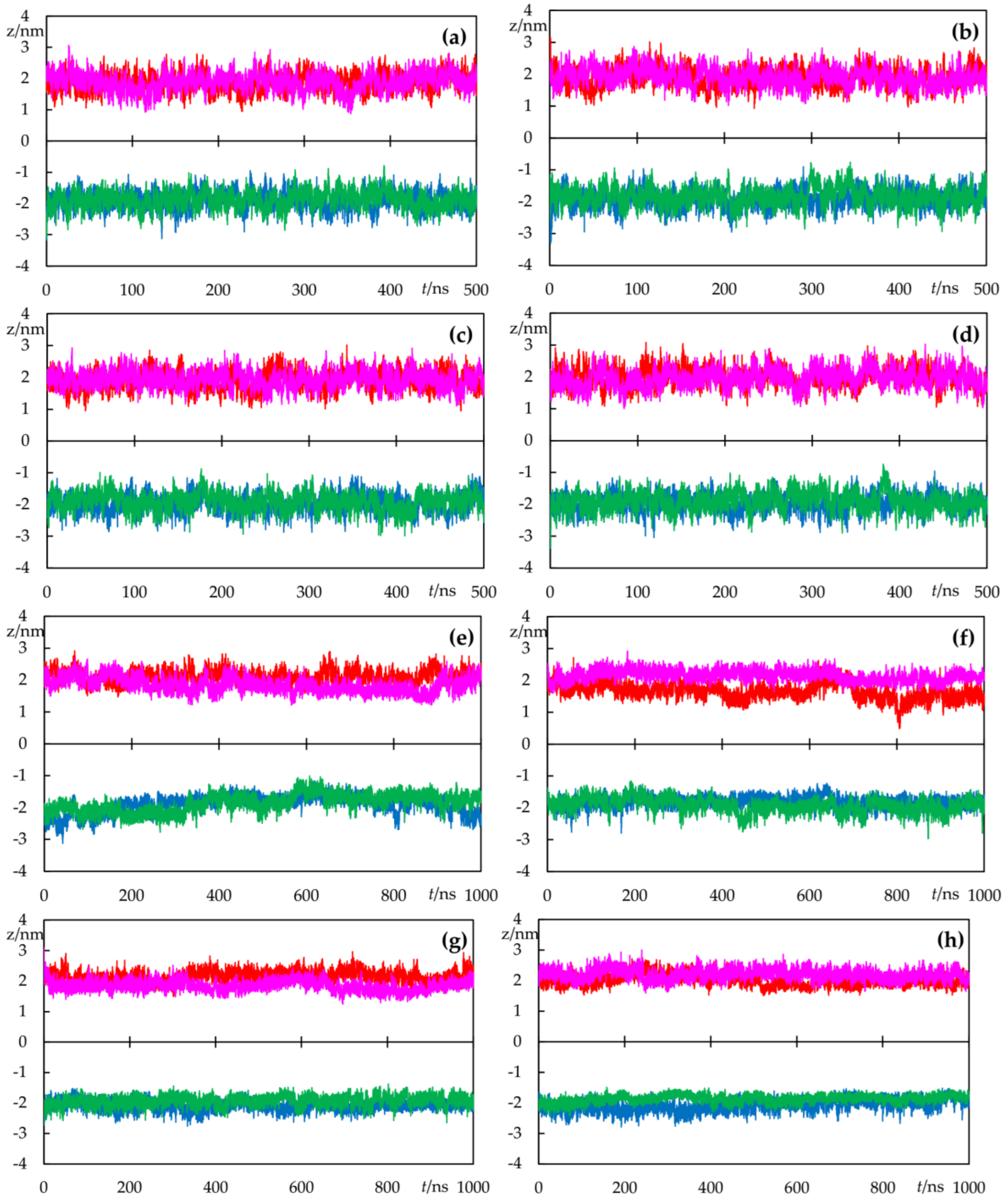

Figure S1 - Time dependence of the positions of the carboxylate group of *c*-PnA (**a**, **b**, **e**, **f**) and *t*-PnA (**c**, **d**, **g**, **h**) in POPC (**a**-**d**) or DPPC (**e**-**f**). Each of the four simulated molecules in each system is depicted in a different color. The two panels in each row pertain to replicate simulations with the same lipid system and probe.

sn-1 acyl chain order parameter profiles in the absence and in the presence of PnA

sn-1 acyl chain order parameter profiles (-S_{CD}; Figure S2) are in line with those observed in the calculation of other related properties, namely area per lipid and bilayer thickness (Figure 3, main text). There are very mild effects on POPC order parameters, most significantly -S_{CD} is slightly increased in the presence of *t*-PnA for the innermost segments, where the rigid linear fluorophore is located. In DPPC, the order parameters are notably increased, especially in the presence of *c*-PnA. The reason for this is the lower tilt of both probes (but especially *c*-PnA), that in turn induce a reduction in the tilt of nearby DPPC acyl chains.

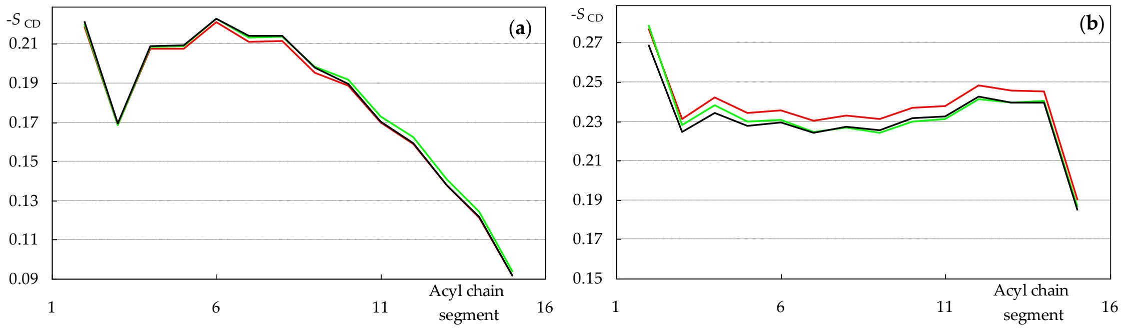

Figure S2 - POPC (**a**) or DPPC (**b**) *sn*-1 acyl chain order parameter profiles for pure lipid (black) or in the presence of *c*-PnA (red) or *t*-PnA (green).

Fully extended radial distribution functions in the POPC simulations

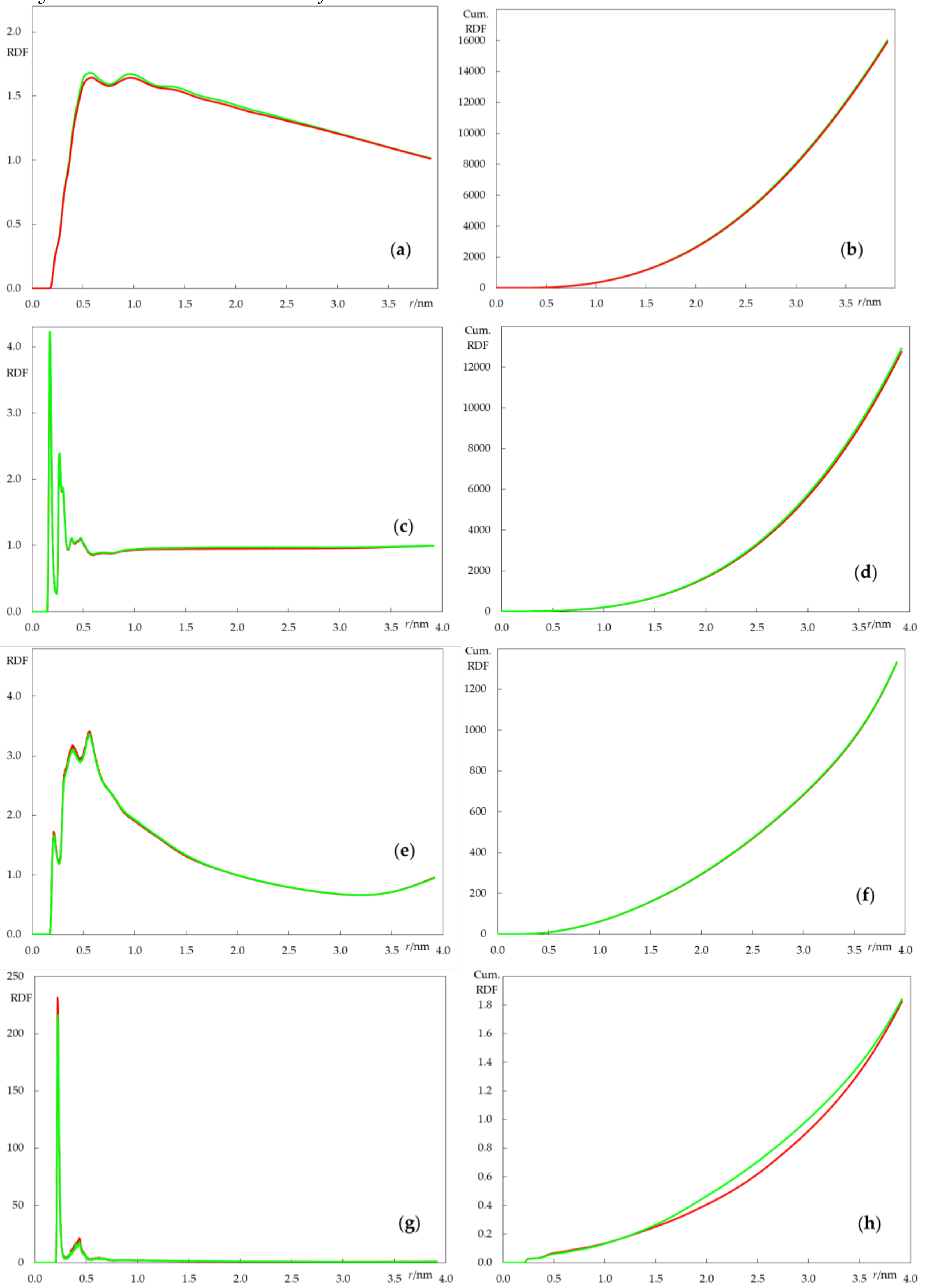

Figure S3 - Fully extended atom-atom radial distribution density functions (RDFs, left plots) and their cumulative counterparts (cumulative RDFs, right plots) of lipid around PnA (**a**, **b**) and water (**c**, **d**), lipid choline (**e**, **f**) and sodium ion (**g**, **h**) around PnA carboxylate, calculated for the POPC simulations. Red and green curves refer to simulations with *c*-PnA and *t*-PnA, respectively.

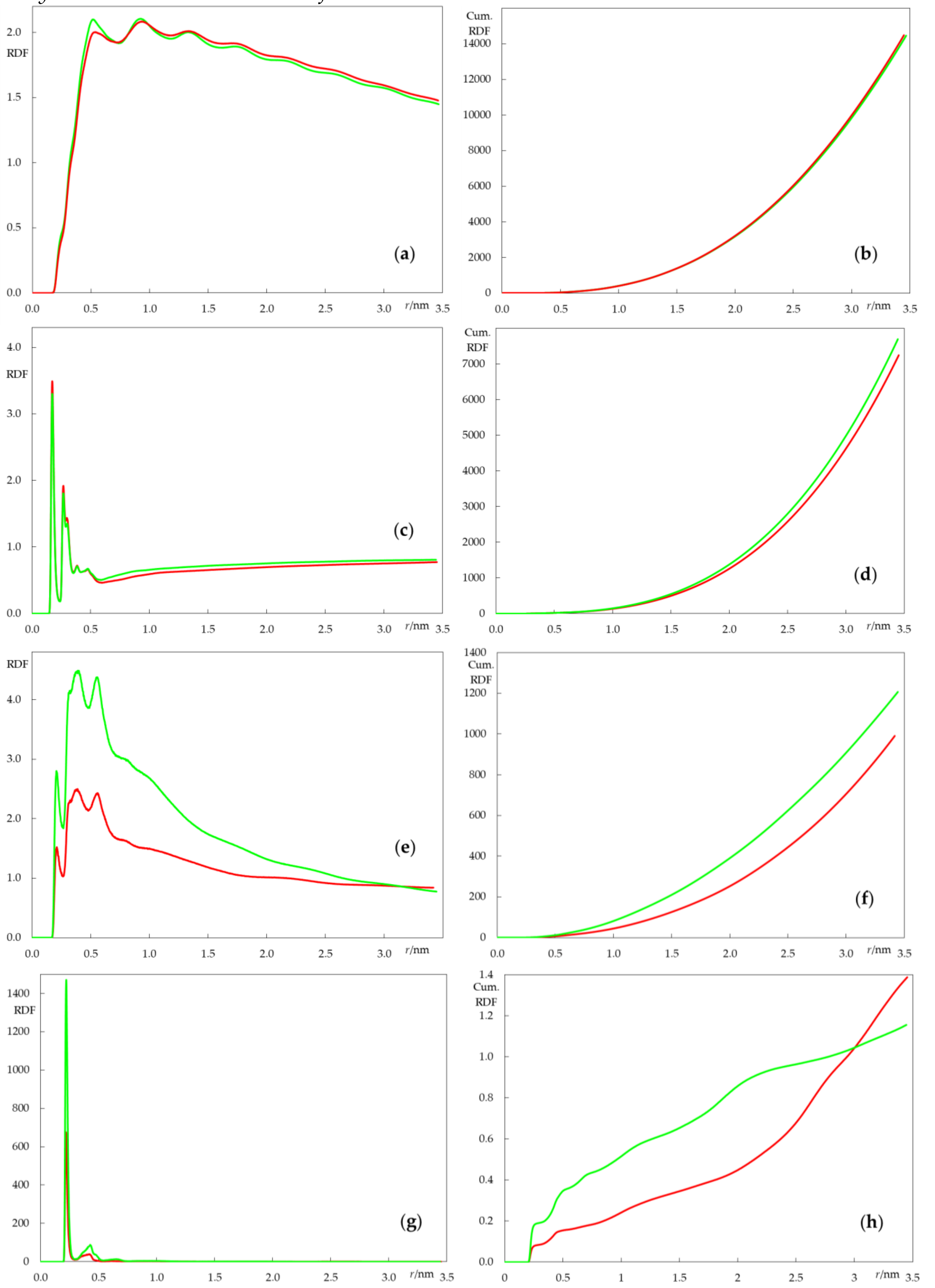

Figure S4 - Fully extended atom-atom radial distribution density functions (RDFs, left plots) and their cumulative counterparts (cumulative RDFs, right plots) of lipid around PnA (**a**, **b**) and water (**c**, **d**), lipid choline (**e**, **f**) and sodium ion (**g**, **h**) around PnA carboxylate, calculated for the DPPC simulations. Red and green curves refer to simulations with c-PnA and t-PnA, respectively.

Time variation of lateral mean square displacements for the POPC and DPPC simulations with inserted c-PnA or t-PnA

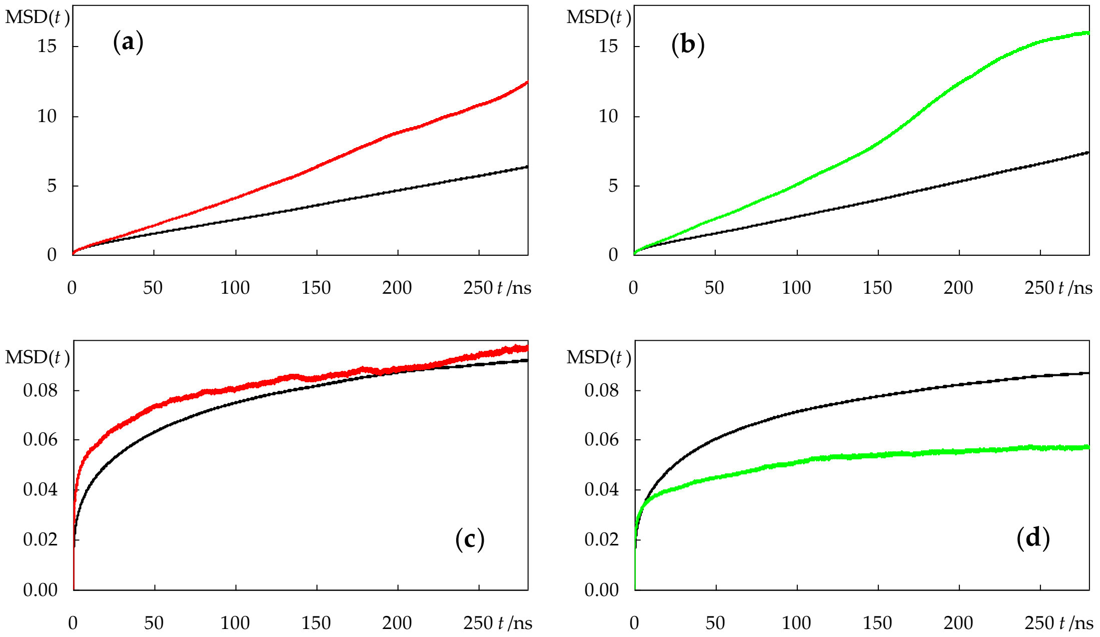

Figure S5 - Time variation of lateral mean square displacements (MSD) for the POPC (**a**, **b**) and DPPC (**c**, **d**) simulations with inserted *c*-PnA (**a**, **c**) or *t*-PnA (**b**, **d**). MSD of the host lipid, *c*-PnA and *t*-PnA are shown as black, red and green curves, respectively.

Free energy profile of PnA across DPPC and POPC bilayers

The PMF profiles for the simulated parinaric acids, spanning the whole bilayer thickness in both POPC and DPPC bilayers are shown in Figure S6.

As can be observed, in some cases there were significant sampling problems on the umbrella sampling simulations, namely in DPPC. As reported previously [1], these problems originate when the pulling of the solute starts in the water, in this case at negative values of the reaction coordinate, as shown in Figures S6b and S6d. To avoid any contribution of this issues in the calculation of free energy barriers, the energy differences calculated in this work are calculated only with the information of the molecule pulled from the center of the bilayer to the water, i.e., from the positive region of the reaction coordinate.

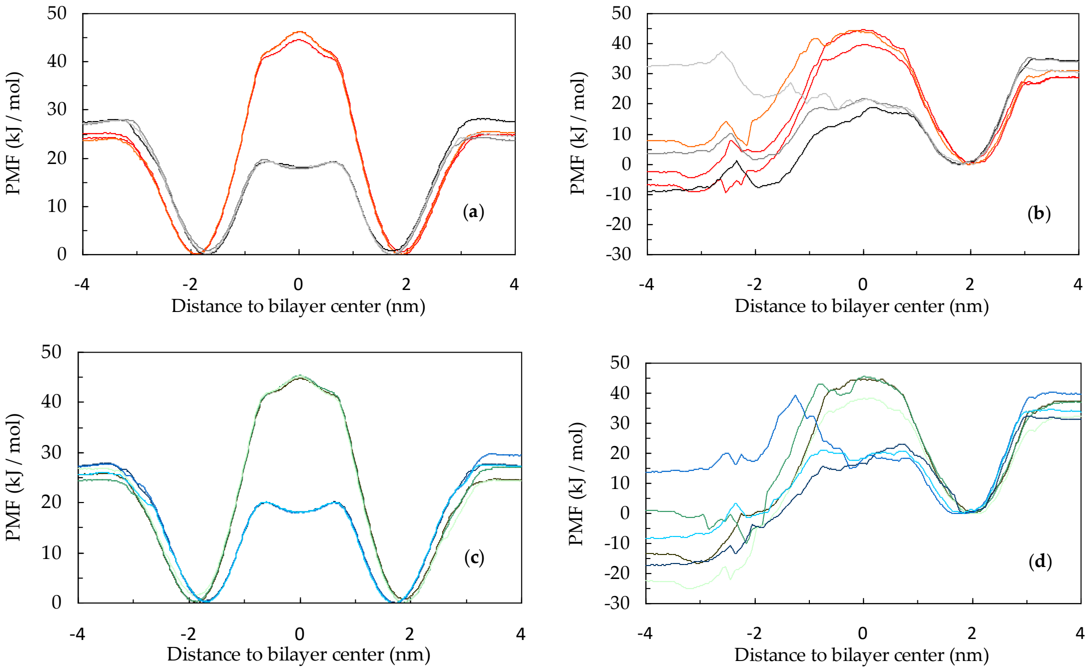

Figure S6 – Potential of mean force (PMF) profiles across the lipid bilayers of *c*-PnA in POPC (**a**), *c*-PnA in DPPC (**b**), *t*-PnA in POPC (**c**), and *t*-PnA in DPPC (**d**). Data are presented with the following color code: ionized *c*-PnA (red), neutral *c*-PnA (black/gray), ionized *t*-PnA (green), and neutral *t*-PnA (blue). Curves with different shades of these colors correspond to profiles obtained from replicate sets of umbrella sampling simulations.

CG parameters for PnA molecules

The CG parametrization of both probes was obtained based on the previous Martini 2.2 parameters used for fatty acids. The simple modifications done are resumed to the attribution of a bead type C4 to the beads involved in the double bonds, and to the angle values to reflect *trans* or *cis* conformations.
The final topologies for *t*-PnA and *c*-PnA are shown below.

<u>Topology for unprotonated *t*-PnA</u>

```
[ moleculetype ]
; molname     nrexcl
  TPA         1

[ atoms ]
; id    type    resnr   residu  atom    cgnr    charge  mass
  1     Qa      1       TPA     COO     1       -1
  2     C1      1       TPA     C1      2        0
  3     C4      1       TPA     D2      3        0
  4     C4      1       TPA     D3      4        0
  5     C4      1       TPA     D4      5        0

[ bonds ]
; i j   funct   length  force.c.
  1 2   1       0.47    1250
  2 3   1       0.47    1250
  3 4   1       0.47    1250
  4 5   1       0.47    1250

[ angles ]
; i j k funct   angle   force.c.
  1 2 3         2       180.0   25.0
  2 3 4         2       180.0   45.0
  3 4 5         2       180.0   45.0
----------------------------------------------------------------------------------------------------
```

<u>Topology for protonated *t*-PnA</u>

```
[ moleculetype ]
; molname     nrexcl
  TPA         1

[ atoms ]
; id    type    resnr   residu  atom    cgnr    charge  mass
  1     P4      1       TPA     COO     1       0
  2     C1      1       TPA     C1      2       0
  3     C4      1       TPA     D2      3       0
  4     C4      1       TPA     D3      4       0
```

```
 5    C4    1    TPA   D4    5     0

[ bonds ]
; i j   funct  length  force.c.
  1  2   1     0.47    1250
  2  3   1     0.47    1250
  3  4   1     0.47    1250
  4  5   1     0.47    1250

[ angles ]
; i j k  funct  angle   force.c.
  1  2  3    2   180.0   25.0
  2  3  4    2   180.0   45.0
  3  4  5    2   180.0   45.0
```

Topology for unprotonated *c*-PnA

```
[ moleculetype ]
; molname    nrexcl
  CPA        1

[ atoms ]
; id   type   resnr   residu  atom   cgnr   charge   mass
  1    Qa     1       CPA     COO    1      -1
  2    C1     1       CPA     C1     2       0
  3    C4     1       CPA     D2     3       0
  4    C4     1       CPA     D3     4       0
  5    C4     1       CPA     D4     5       0

[ bonds ]
; i j   funct  length  force.c.
  1  2   1     0.47    1250
  2  3   1     0.47    1250
  3  4   1     0.47    1250
  4  5   1     0.47    1250

[ angles ]
; i j k  funct  angle   force.c.
  1  2  3    2   180.0   25.0
  2  3  4    2   120.0   45.0
  3  4  5    2   120.0   45.0
```

Topology for protonated *c*-PnA

```
[ moleculetype ]
; molname    nrexcl
  CPA        1
```

```
[ atoms ]
; id    type    resnr   residu  atom    cgnr    charge  mass
  1     P4      1       CPA     COO     1       0
  2     C1      1       CPA     C1      2       0
  3     C4      1       CPA     D2      3       0
  4     C4      1       CPA     D3      4       0
  5     C4      1       CPA     D4      5       0

[ bonds ]
; i j   funct   length  force.c.
  1  2  1       0.47    1250
  2  3  1       0.47    1250
  3  4  1       0.47    1250
  4  5  1       0.47    1250

[ angles ]
; i j k funct   angle   force.c.
  1  2  3       2       180.0   25.0
  2  3  4       2       120.0   45.0
  3  4  5       2       120.0   45.0
----------------------------------------------------------------------------------------------------------
```

Comparison of bond length and angle distributions for the Martini and atomistic mapped parameterization of PnA molecules

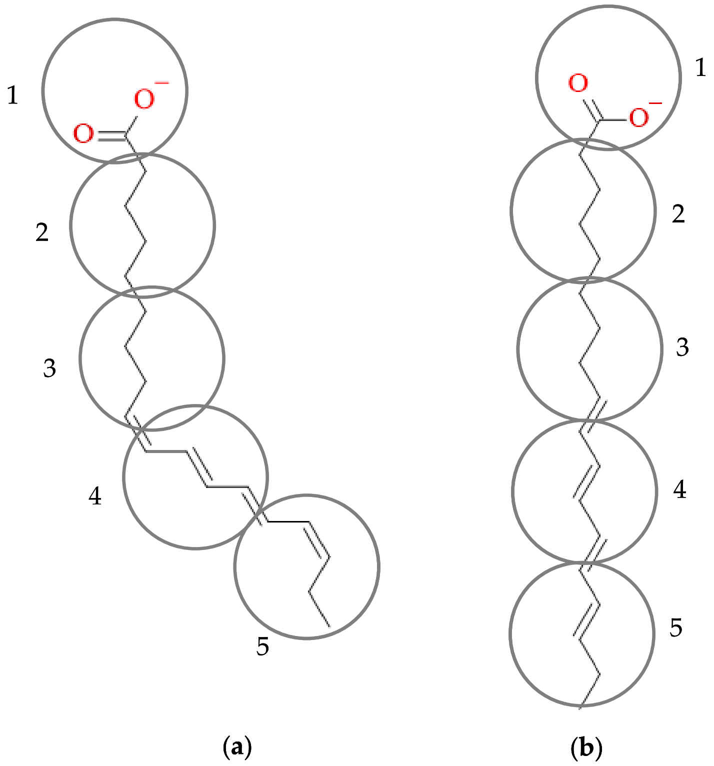

Figure S7 –CG mapping scheme for *c*-PnA (**a**) and *t*-PnA (**b**).

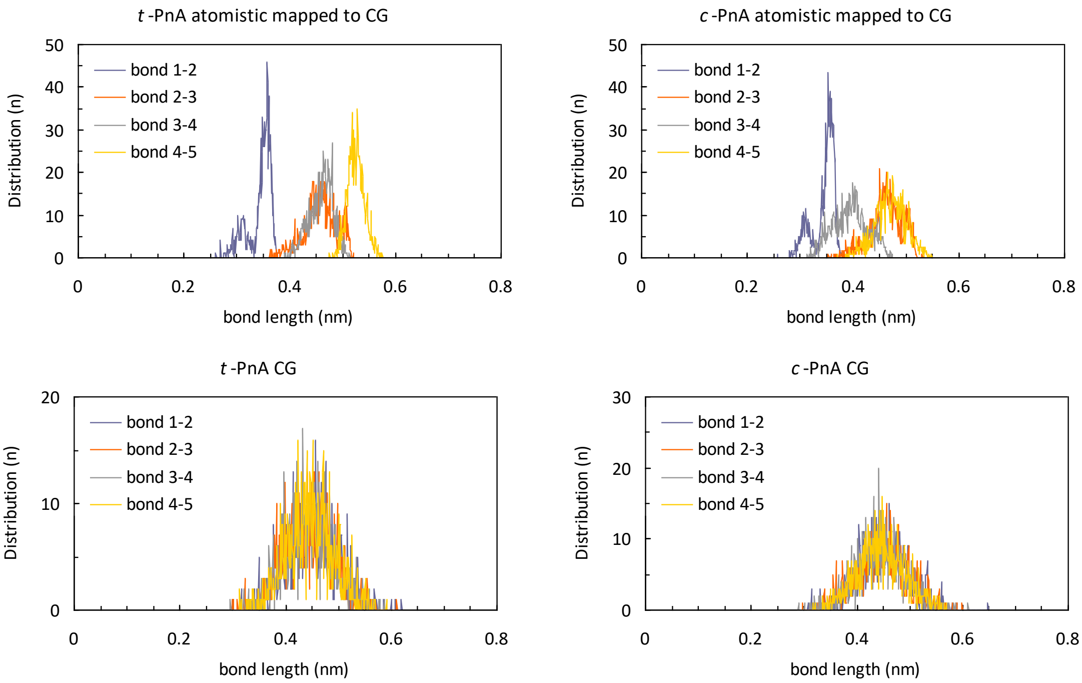

Figure S8 – Comparison of the atomistic and CG mapping for the different CG bonds.

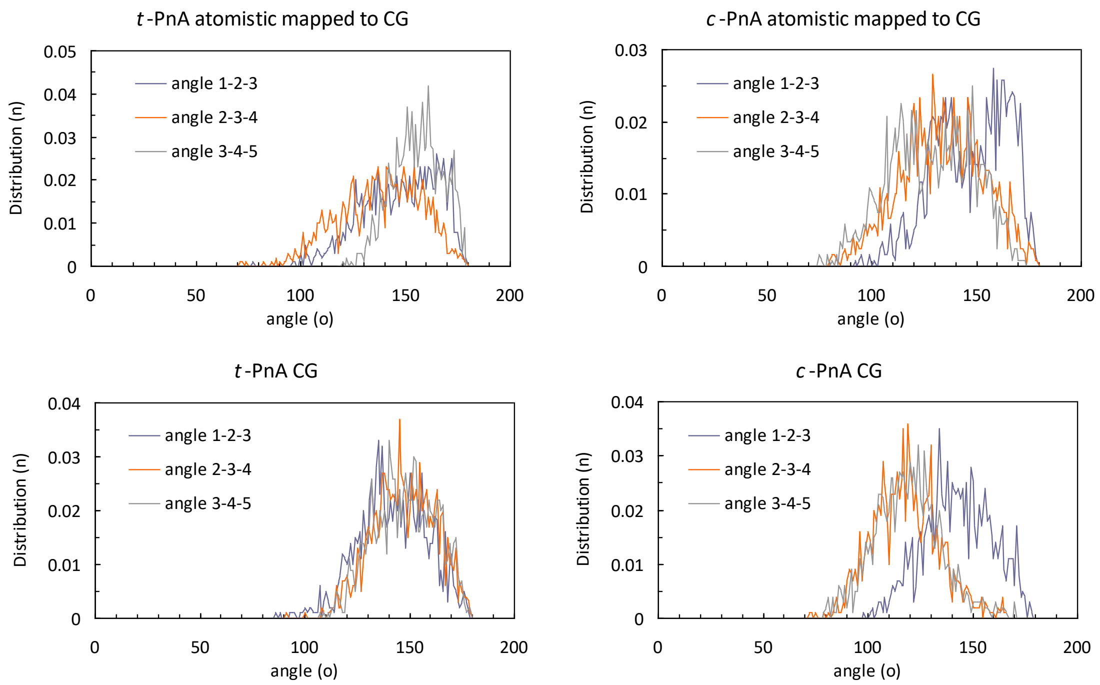

Figure S9 – Comparison of the atomistic and CG mapping for the different CG angles.

Snapshots of POPC and DPPC membranes used in the calculation of PMF profiles

Figure S10 shows the snapshot of POPC and DPPC membranes used in the calculation of PMF profiles, after 10 μs of simulation, at 298K. Consistent with its gel state, the DPPC membrane is thicker than POPC membrane in the liquid disordered state. Apparent from the observation of the snapshots is the less thick water layer in POPC, which is a visualization effect due to the larger area/lipid and consequently lager box area in POPC compared to the DPPC membrane, while conserving the number of lipids and water beads in the system.

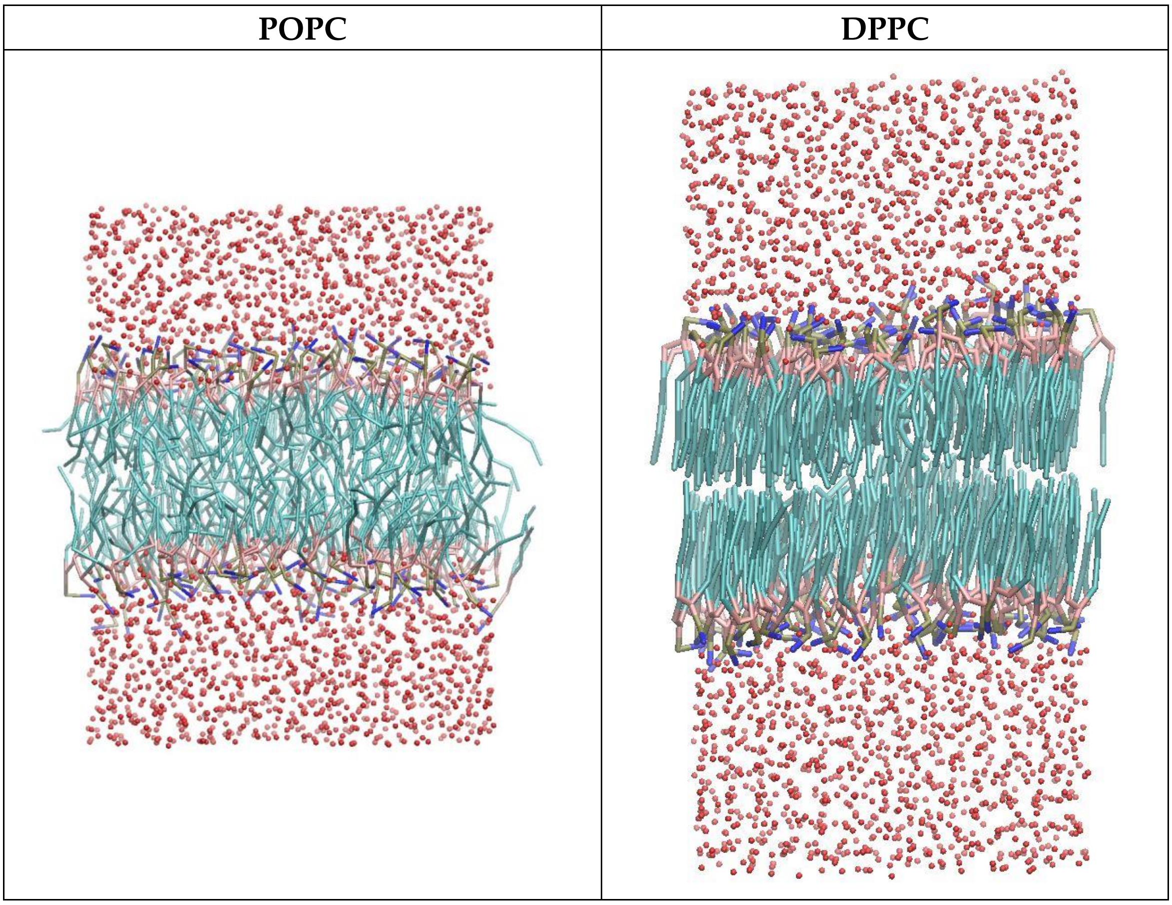

Figure S10 - Snapshot of POPC (left) and DPPC (right) membranes used in the calculation of PMF profiles, after 10 μs of simulation, at 298 K.

Reference

1. Filipe, H.A.L.; Moreno, M.J.; Róg, T.; Vattulainen, I.; Loura, L.M.S. How To Tackle the Issues in Free Energy Simulations of Long Amphiphiles Interacting with Lipid Membranes: Convergence and Local Membrane Deformations. *J. Phys. Chem. B* **2014**, *118*, 3572–3581, doi:10.1021/jp501622d.
